# Sequence-based artificial intelligence-guided inhibitory peptides to counteract AAV neutralizing antibodies in gene therapy

**DOI:** 10.1016/j.omta.2026.201832

**Published:** 2026-08-06

**Authors:** Bingqi Xu, Xin Zhang, Dandan Yu, Panjing Wang, Ying Chi, Wei Liu, Feng Xue, Lingling Chen, Huiyuan Li, Wentian Wang, Yunfei Chen, Xiaofan Liu, Rongfeng Fu, Renchi Yang, Wenwei Shao, Siwei Yu, Xiaolei Pei, Lei Zhang

**Affiliations:** 1 State Key Laboratory of Experimental Hematology, National Clinical Research Center for Blood Diseases, Haihe Laboratory of Cell Ecosystem, Institute of Hematology & Blood Diseases Hospital, Chinese Academy of Medical Sciences & Peking Union Medical College, Tianjin 300020, P.R. China; 2 Tianjin Institutes of Health Science, Tianjin 300020, P.R. China; 3 Harbin Institute of Technology, Department of Mathematics and Institute of Artificial Intelligence, Harbin 150001, P.R. China; 4 Academy of Medical Engineering and Translational Medicine, Tianjin University, Tianjin 300072, P.R. China

**Keywords:** adeno-associated virus, neutralizing antibodies, artificial intelligence, decoy peptides, secondary dosing

## Abstract

Adeno-associated virus (AAV) vectors have demonstrated strong clinical efficacy across multiple monogenic disorders, yet neutralizing antibodies (NAbs) remain major barriers to vector redosing and durable transduction. To address this limitation, we developed a sequence-based artificial intelligence framework integrating Bidirectional Encoder Representations from Transformers (BERT) and Evolutionary Scale Modeling with Low-Rank Adaptation (ESM-LoRA) to identify human-tolerant, virus-specific motifs across human and viral proteins using sliding-window analyses and multi-layer fine-tuning. Using capsid-derived segments from AAV2 and AAV843, we prioritized surface-accessible, virus-polarized peptides as candidate decoys and evaluated their ability to block AAV-targeted antibodies. The models achieved high accuracy in distinguishing human versus viral fragments, and selected peptides bound AAV-specific antibodies with nanomolar affinity, competitively restored capsid binding, and recovered >60% of baseline transduction in the presence of NAbs. In mouse models with pre-existing or redosing-induced antibodies, coadministration of decoy peptides and an IgG-degrading enzyme reduced high-titer NAbs to approximately 1:4 and restored hepatic transgene expression without detectable inflammatory or peptide-specific immune responses. A contrastive variational autoencoder (VAE) further generated non-natural peptides with broader epitope coverage. These results support sequence-driven AI design of short, low-immunogenicity decoy peptides as a translational strategy to mitigate NAb interference and enhance the feasibility of AAV redosing.

## Graphical abstract

**Figure:**
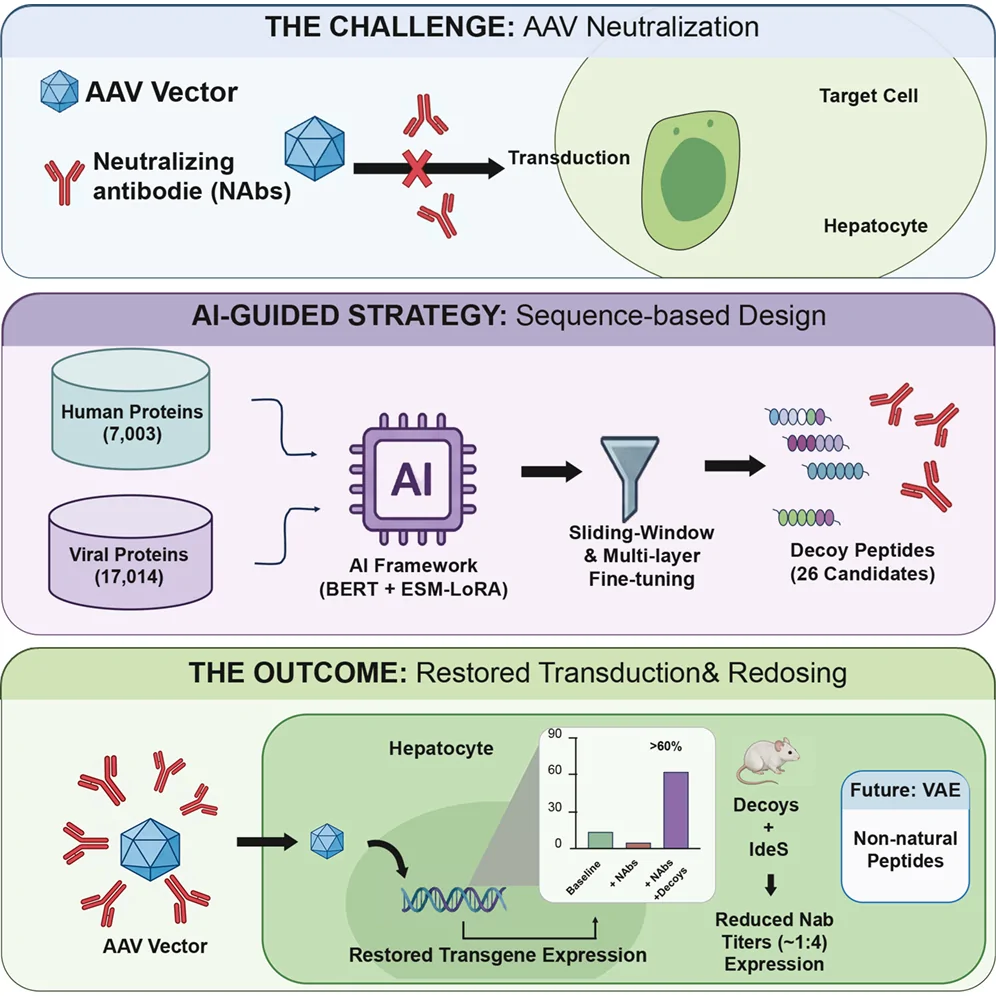

## Teaser

Xu and colleagues develop a sequence-guided peptide decoy strategy to transiently suppress neutralizing antibodies against AAV. By engaging circulating antibodies prior to vector administration, these peptides enable partial restoration of AAV transduction *in vitro* and *in vivo*. This approach provides a potential avenue to expand patient eligibility and facilitate repeat dosing in AAV-based gene therapies.

## Introduction

Adeno-associated virus (AAV) vectors have enabled transformative gene therapies for multiple monogenic disorders. However, neutralizing antibodies (NAbs) present in patient plasma remain a major barrier, limiting efficient vector delivery and long-term therapeutic success.^1^^,^^2^ Systemic AAV administration, as commonly used for disorders such as hemophilia,^3^^,^^4^^,^^5^^,^^6^ glycogen storage disease,^7^ and Duchenne muscular dystrophy^8^ is more frequently associated with the development of NAb responses. In contrast, locally administered therapies, including those targeting retinal and auditory disorders,^9^^,^^10^ tend to be less affected by circulating NAbs. Nearly all patients develop NAbs following AAV gene therapy, which can persist at high titers for years,^11^ representing the major obstacle to vector redosing.^12^

Current approaches to overcoming NAb barriers remain limited in efficacy and applicability. Empty AAV capsids can serve as decoys^13^ but trigger additional immune responses; IgG-degrading enzymes (IdeS) reduce NAb titers in nonhuman primates^14^ yet face challenges of immunogenicity and limited efficacy against high-titer antibodies; and AAV encapsulation within nanovesicles or lipid nanoparticles^15^ may shield virions from circulating antibodies but may also alter masking tropism and introducing new immunogenic barriers.

Evidence from autoimmune disease research highlights an alternative path: peptide-based antibody decoys. In lupus models, D-peptides delivered via nanoparticles prolonged renal exposure and alleviated nephritis^16^ and mimotopes such as DWEYS competitively neutralized pathogenic cross-reactive antibodies.^17^ These findings underscore the unique advantages of peptides, including combined linear and conformational epitope presentation, high specificity, low toxicity, and ease of design.^18^^,^^19^^,^^20^ This reciprocal recognition provides a mechanistic rationale for using short peptides as decoys to effectively block NAbs binding to AAV capsids.

Recent advances in natural language processing (NLP) have been successfully applied to protein research,^21^ with transformer-based models achieving strong performance in structure prediction, feature extraction, and sequence generation.^22^^,^^23^^,^^24^^,^^25^ Leveraging the principle that the immune system tolerates self-proteins but reacts to pathogen-derived proteins, sequence-based artificial intelligence (AI) approaches^26^^,^^27^ provide a powerful framework to identify and optimize immunogenic regions.

Here, we developed sequence-based AI models to identify short AAV-derived peptides capable of acting as NAb decoys. We hypothesized that peptides located within the cumulative and peak regions of the viral-scoring profile represent the most promising decoy candidates, a hypothesis that was subsequently tested through extensive experimental assays. We validated their ability to block both pre-existing and therapy-induced antibodies, demonstrated inhibitory efficacy in hemophilia B (HB) patients after AAV gene therapy, and confirmed therapeutic benefit in *in vivo* models. Finally, we employed generative AI strategies to design novel peptides with potent inhibitory effects. These findings highlight AI-guided peptide engineering as a translational strategy to overcome NAb barriers and broaden the clinical applicability of AAV gene therapy.

## Results

### Sequence-based models distinguish human-derived from virus-derived short peptides

**Figure 1:**
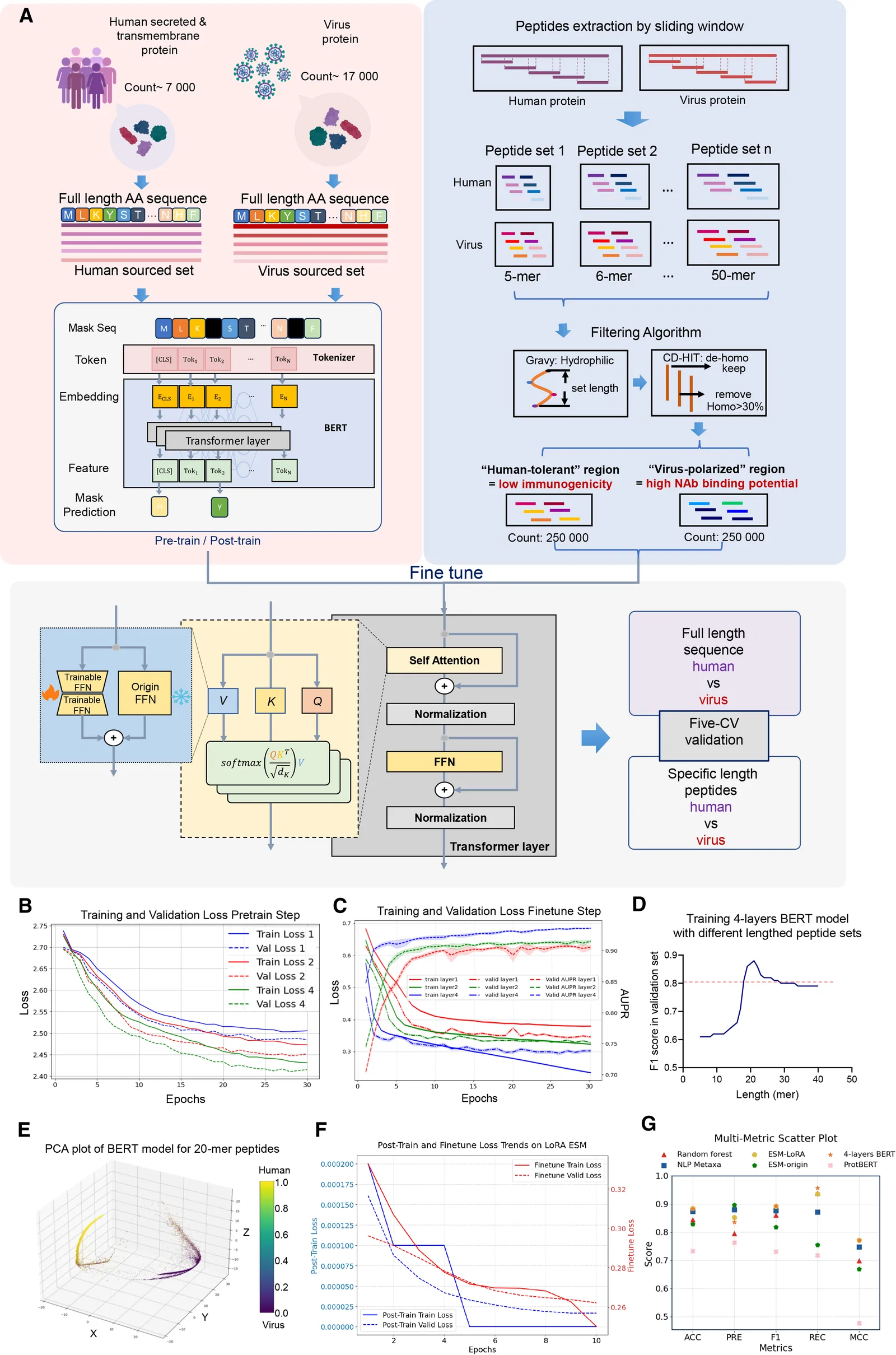
Sequence-based BERT framework for classifying human-versus virus-derived peptides (A) Workflow for dataset construction, including compilation of human and viral proteins, sliding-window peptide generation, hydrophilicity filtering, and model development using BERT architectures. (B and C) Training performance of shallow BERT models (one, two, or four layers). (D) Classification performance across peptide lengths (5–50 amino acids). (E) Principal component analysis (PCA) of embeddings shows clear separation between human-derived and virus-derived 20-mer peptides. (F) Post-training and fine-tuning loss curves for the LoRA-adapted ESM-2 model. (G) Comparative held-out validation folds of fine-tuned BERT models and baseline classifiers using 5-fold cross-validation, demonstrating improved stability and accuracy.

To guide decoy peptide design, we aimed to identify peptide sequences enriched in viral proteins while being distinct from human proteins, thereby increasing the likelihood of targeting antibody-recognized viral epitopes. We sought to determine whether sequence-based models can distinguish human-derived from virus-derived short peptides, providing a basis for decoy design. We compiled 7,003 human membrane/secreted proteins and 17,014 viral proteins from UniProtKB. Sliding windows (lengths 5–50 amino acids, a one-residue step) generated peptide candidates; to prioritize soluble and experimentally tractable candidates, we retained for hydrophilic peptides (GRAVY < −1.0) (Figure 1A). Given that our primary task involves classification and ranking of short peptide sequences rather than de novo sequence generation, we selected Bidirectional Encoder Representations from Transformers (BERT) as a computationally efficient and well-established architecture for sequence classification. More complex generative models, such as diffusion-based approaches, were not used for the primary classification task but were partially addressed through our variational autoencoder (VAE)-based peptide generation framework.

We compared shallow BERT classifiers (one, two, or four layers; pre-trained on full-length proteins and fine-tuned on peptides) with a parameter-efficient low-rank adaptation (LoRA)-adapted Evolutionary Scale Modeling-2 (ESM2, 33 layers). Models were trained and evaluated using protein-disjoint fivefold cross-validation, with all peptide windows derived from the same source protein assigned to the same fold (Figures 1B and 1C). Performance peaked for peptide lengths between 18 and 25 amino acids, with F1 scores consistently exceeding 0.80 (Figure 1D). We therefore selected 20-mer peptides for downstream analyses as a balance between model performance and biological interpretability and did not imply that this length is optimal for decoy activity. Visualization of the learned embeddings showed clear separation between human- and virus-derived 20-mer peptides (Figure 1E). Fine-tuned BERT and LoRA-ESM2 outperformed ProtBERT, Random Forest, and NLP-Metaxa baselines on the held-out folds (Figures 1F and 1G). To reduce potential hydrophilicity confounding, we introduced a third “hydrophobic” label during fine-tuning, and the models maintained robust three-class discrimination (Figure S1).

Collectively, peptide polarization was defined based on the predicted class probability from the trained classifier, where higher viral-class probability indicates virus-polarized peptides and higher human-class probability indicates human-polarized peptides.

### AI-guided classification identifies virus-polarized, human-polarized, and hydrophobic peptides across 11 AAV capsids

**Figure 2:**
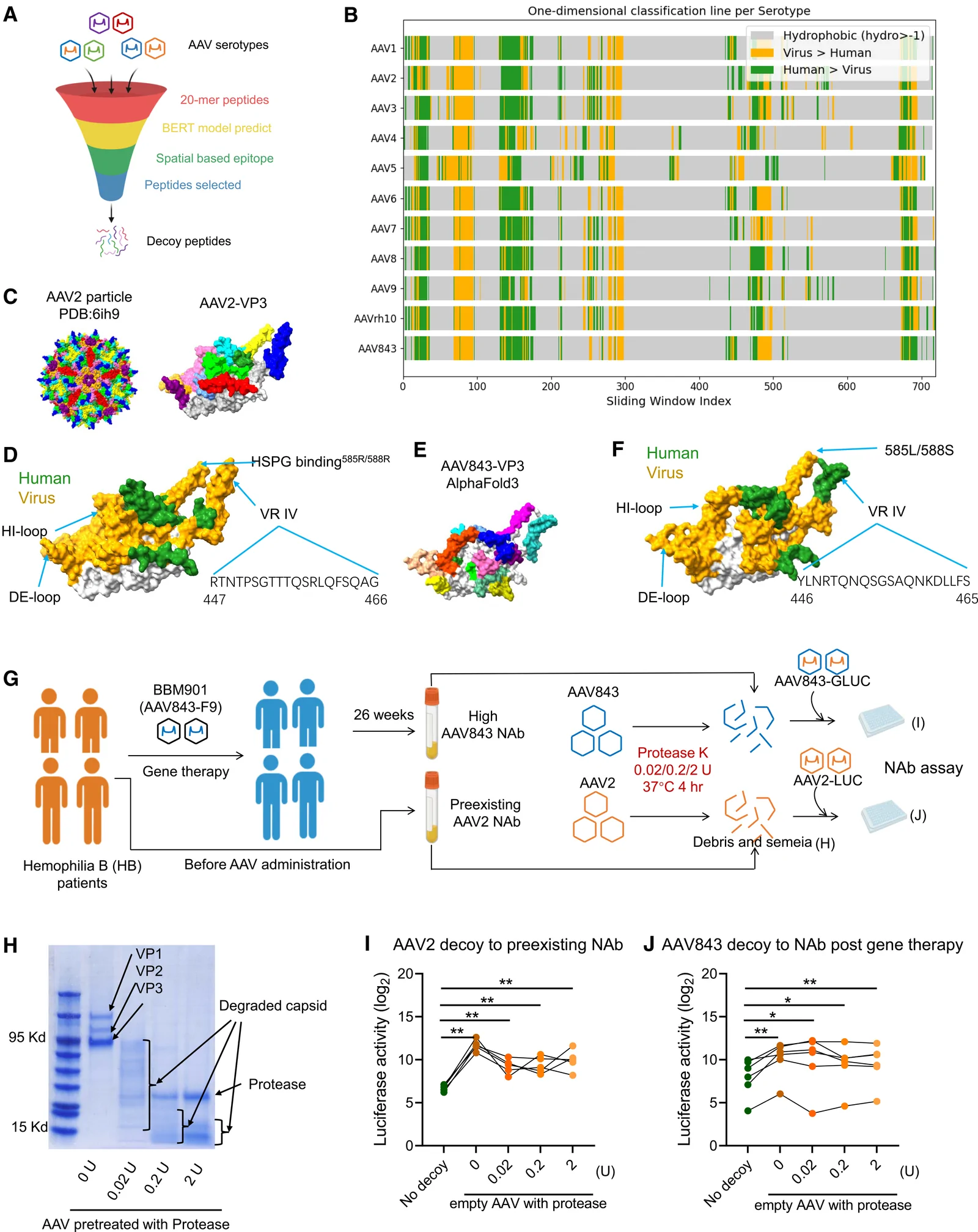
BERT-based identification and structural mapping of virus- and human-polarized peptides across AAV capsids (A) Workflow illustrating the use of fine-tuned BERT models to classify short peptides as virus- or human-polarized, followed by structural mapping and candidate decoy selection. (B) Predicted polarity distribution of 20-mer peptides across the capsid proteins of 10 common AAV serotypes and the AAV843 variant (Table S1). (C–F) Structural mapping of polarity predictions onto capsid models of AAV2 (C and D, PDB 6IH96ih9) and AAV843 (E and F, AlphaFold 3 prediction). Virus-polarized peptides are highlighted in yellow and human-polarized peptides in green. (G) NAb titers in plasma from hemophilia B patients before AAV gene therapy (*n* = 5) and 26 weeks after BBM901 treatment (*n* = 5), evaluated for inhibition by intact empty capsids or by protease-derived capsid fragments. (H) Proteinase K digestion of AAV2 empty capsids (1 μg) at increasing enzyme amounts for 4 h at 37°C, with degradation confirmed by SDS-PAGE. (I and J) NAb inhibition assays showing by luciferase (Luc) activity that protease-generated fragments from AAV2 (I) or AAV843 (J) selectively neutralized patient plasma with serotype-specific antibodies.

We next applied the fine-tuned BERT classifier to classify short peptides derived from AAV capsids (Figure 2A). Using capsid protein sequences from 10 common AAV serotypes and the AAV843 variant (used in BBM901, an AAV843-factor IX (FIX) gene therapy vector for hemophilia B^4^), we assigned model-predicted class probabilities, classifying them as virus-polarized, human-polarized, or hydrophobic (Figure 2B; Table S1). Polarization scores represent the model-predicted likelihood that a peptide resembles human versus viral sequence distributions, rather than indicating biological origin or immunogenicity. Differences in polarization across serotypes did not directly incorporate structural position.

Although peptides are linear, several adopt surface-loop conformations overlapping with known AAV2-NAb interfaces. To visualize their distribution, polarity predictions were mapped onto structural models of AAV2 (cryo-EM, PDB: 6IH9) and AAV843 (AlphaFold3 prediction). Distinct clusters of virus versus human-polarized peptides were observed across capsid surfaces (Figures 2C–2F). Notably, both virus- and human-polarized peptides were distributed on surface-exposed regions, indicating that surface accessibility alone does not distinguish antibody-binding sites.

Instead, polarization scores were used as a prioritization metric to identify regions enriched in viral sequence features, which are less similar to host-derived sequences. These candidate regions were subsequently evaluated in combination with structural context and experimental validation.

To assess the clinical relevance of these predictions, plasma samples were collected from patients with hemophilia B. NAbs against AAV2 were assessed using plasma containing naturally occurring pre-existing immunity, while antibodies against AAV843 were evaluated using plasma from patients following AAV843 gene therapy. These assays were conducted independently and reflect distinct antibody populations (Figure 2G). When AAV2 or AAV843 empty capsids were digested with protease, the resulting peptide fragments selectively inhibited NAbs in a serotype-specific manner (Figures 2H–2J). These findings suggest that, in addition to intact capsids, degradation fragments may also contribute to neutralizing antibody inhibition, although with lower efficiency.

### Functional validation of AAV2- and AAV843-derived decoy peptides

The AI framework was used to prioritize candidate peptides by reducing the search space, rather than to directly predict functional decoy activity. Furthermore, cumulative scoring based on model-predicted polarity was used to further refine candidate selection. While the original goal included identifying human-tolerant features, in this study we focused on viral-enriched peptide regions as candidates for antibody interaction, with experimental validation used to determine functional activity.

**Figure 3:**
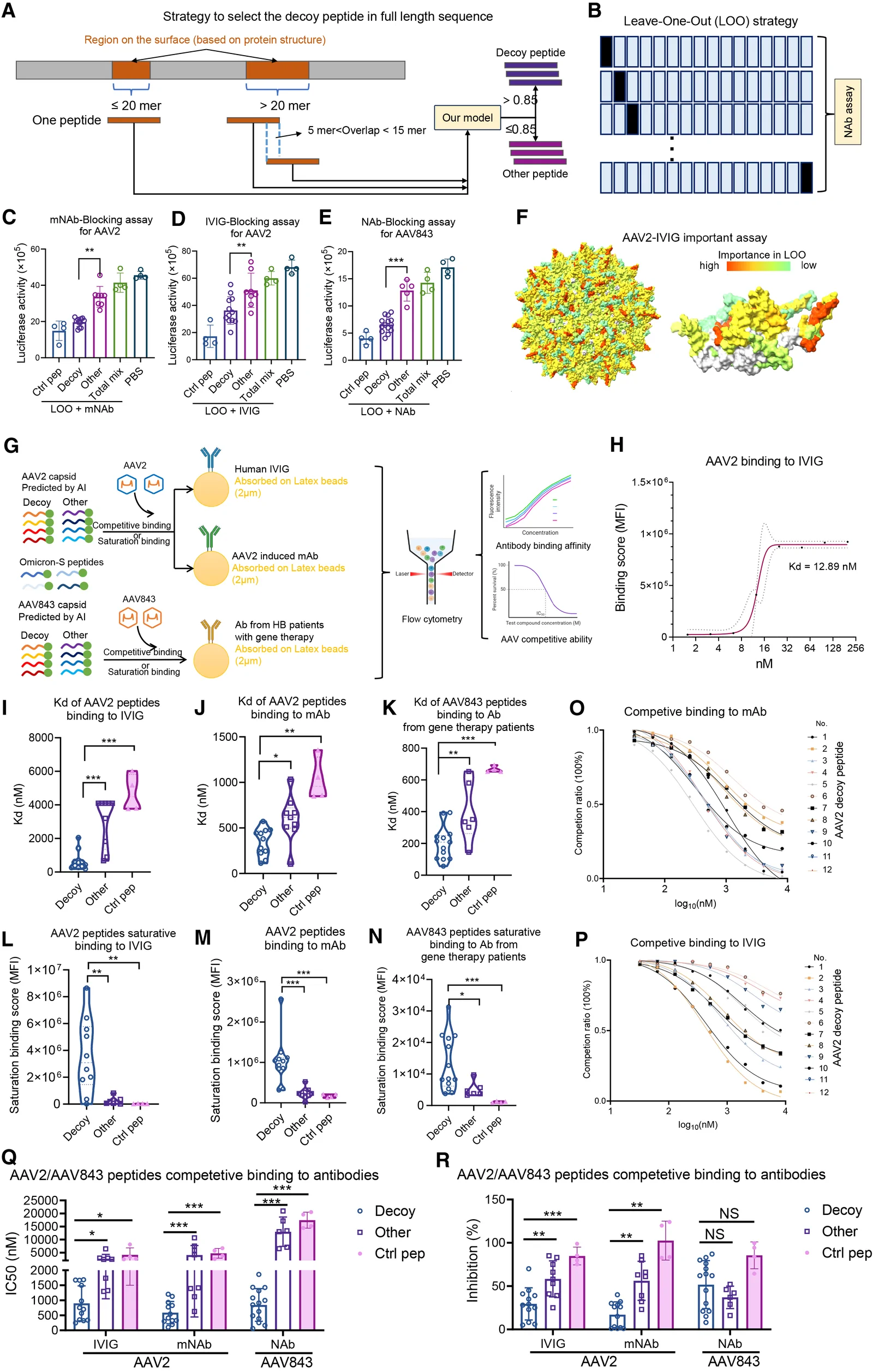
Functional validation of BERT-prioritized decoy peptides through LOO inhibition assays, direct binding, and competitive binding analyses (A) Workflow for candidate peptide selection from AAV2 and AAV843 capsids, integrating BERT-based polarity predictions with structural mapping. (B) LOO peptide-omission assay design: each peptide mixture contained all peptides except one (10 μM final concentration). (C–E) LOO assays assessing NAb inhibition in AAV2 NAbs in plasma from immunized mice, human IVIG, and plasma from BBM901-treated patients. Each data point represents the mean of three technical replicates after omission of a single peptide from the mixture. reporter activity was measured in HEK293 cells following AAV2-Luc or AAV843-Gluc transduction. (F) Structural mapping of selected AAV2 decoy peptides onto capsid surfaces. (G) Experimental design for microsphere-based binding and competitive assays. (H) Saturation binding curves of biotin-labeled AAV2 particles (1.56–200 nM) with IVIG-coated microspheres. (I–N) Binding affinities (Kd) and maximal binding signals measured as MFI of decoy versus control peptides, measured with IVIG (I) and (L), mNAbs (J) and (M), and immunoglobulins from BBM901-treated patients (K), and (N). (O–P) Competitive binding assays: biotin-labeled AAV2 particles (200 nM) were incubated with serially diluted peptides (0–8,192 nM) and antibody-coated microspheres. Competitive binding curves were generated, and IC50 values were calculated. The numbers (1–12) represent individual peptide candidates. (Q) AAV2 decoys versus AAV2 particles with IVIG and mNAbs, and AAV843 decoys versus AAV843 particles with patient-derived Ig. lower IC_50 values indicate stronger competitive ability. (R) Competitive inhibition assay measuring binding of biotin-labeled AAV2 or AAV843 particles to antibody-coated beads in the presence of increasing concentrations of peptides. Binding was detected using PE-Streptavidin, and inhibition percentages were calculated.

To functionally validate the BERT-prioritized epitopes, we applied a sliding-window strategy (fixed window length of 20 amino acids) combined with polarity-based ranking to the capsid proteins of AAV2 and AAV843 (Figure 3A). This workflow generated over 1,400 peptide candidates, from which 39 short peptides from the VP3 region were prioritized. Peptides overlapping virus-polarized regions were defined as candidate decoys, whereas the remaining peptides served as controls (Table S2).

We first used a leave-one-out (LOO) peptide masking assay to assess each peptide’s contribution to NAb inhibition (Figure 3B). In mixtures containing all but one peptide, removal of specific decoys reduced inhibition of both mouse-derived NAbs to AAV2 (mNAbs, referring to polyclonal antibodies present in mouse plasma) and human intravenous immunoglobulin (IVIG), as indicated by lower luciferase (Luc) expression (Figures 3C and 3D). Given the polyclonal nature of NAbs, removal of a single peptide in the LOO assay is not expected to produce a large change in overall neutralization, as multiple peptides contribute redundantly to antibody binding. Notably, the critical peptides differed between mNAbs and IVIG, suggesting source-specific epitope preferences (Figure S2). Application of the LOO assay to AAV843-derived peptides further identified peptides whose omission reduced protection against NAbs in plasma from BBM901-treated patients (Figures 3E and S2). Structural mapping localized these peptides to exposed capsid regions, reinforcing their relevance in antibody recognition (Figures 3F and S3).

We next tested direct peptide-antibody interactions using a microsphere-based binding assay (Figures 3G and 3H). To assess specificity, we included four control peptides derived from the Omicron spike protein. Across both mNAbs and IVIG, decoy peptides exhibited significantly apparent dissociation constants (Kd) and higher binding saturation levels compared to controls, indicating stronger and greater maximal binding signals (Figures 3I–3N, S4, and S6). In parallel, binding assays using an unrelated monoclonal antibody (anti-PD-1) revealed markedly reduced affinity, further supporting the specificity of peptide-antibody interactions (Figure S6).

Finally, competitive binding assays demonstrated that decoy peptides effectively outcompeted intact AAV capsids for antibody binding. AAV2-derived decoys showed markedly lower half-maximal inhibitory concentrations (IC50) and higher maximal inhibition rates relative to controls (Figures 3O, 3P, S7, and S9). To further quantify these effects, we compared IC50 values across different antibody sources (Figure 3Q). AAV2-derived decoys exhibited stronger competitive binding against IVIG and mNAbs, as well as AAV843 decoys to inhibition of BBM901 treated patient-derived Ig (Figure 3R).

The modest differences observed between decoy and other peptide groups reflect the redundancy of antibody recognition across multiple epitopes, rather than a lack of specificity in the selection strategy. These results indicate that AI-prioritized decoy peptides contribute to NAb inhibition, show preferential binding to patient- and mouse-derived antibodies, and can functionally reduce antibody-mediated interference with AAV transduction.

### Decoy peptide combinations inhibit NAbs *in vitro*

**Figure 4:**
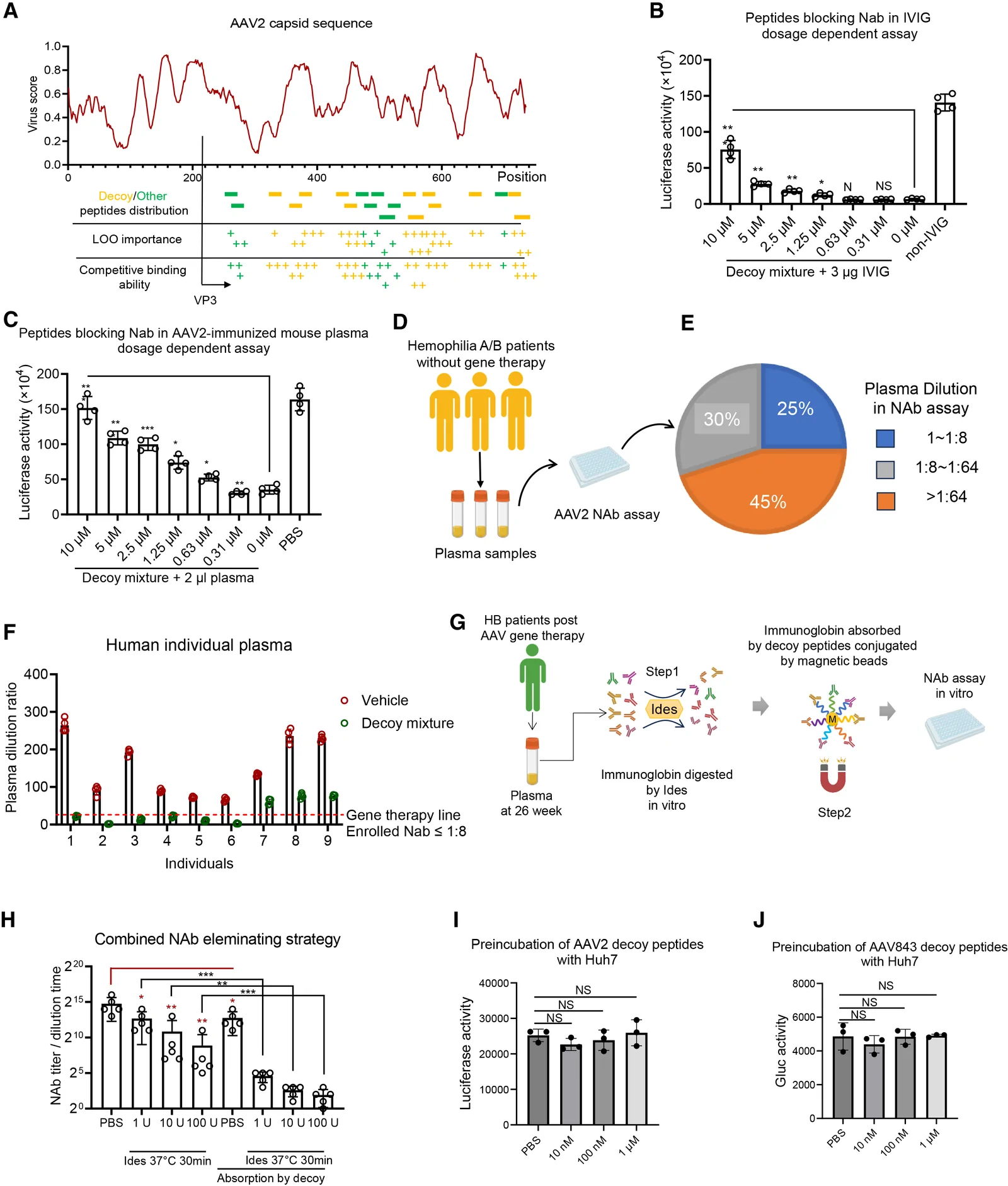
AI-guided decoy peptide mixtures inhibit NAbs *in vitro* (A) Summary of decoy peptide identification from BERT predictions, structural mapping, and functional validation. (B and C) For *in vitro* NAb inhibition, various concentrations of the decoy mixture were incubated with IVIG (3 μg) or mNAbs (2 μL, titer >1:1,024), followed by AAV-Luc transduction. (D and E) Plasma from 20 hemophilia A/B patients was tested for pre-existing AAV2 NAbs. (F) Effect of decoy peptide preincubation on neutralizing antibody titers in selected patient samples. (G and H) Treatment of plasma from BBM901-treated patients with IdeS and AAV843 decoy peptides. In (H), red asterisks denote comparisons between the IdeS-only and PBS groups, and black asterisks denote comparisons between the IdeS-plus-peptide group and the peptide-only group. Statistical significance is denoted as ns, not significant; ∗*p* < 0.05, ∗∗*p* < 0.01, ∗∗∗*p* < 0.001. (I and J) Huh7 cells (2 × 10⁵) were pre-incubated with decoy peptide mixtures at low, medium, and high concentrations, followed by transduction with AAV2-Luc or AAV843-Gluc. Reporter activity was measured 48 h later and compared to PBS-treated controls.

To maximize inhibitory efficacy, validated decoy peptides were pooled in equal proportions to generate a decoy mixture (Figure 4A). *In vitro* assays showed that this mixture restored Luc activity to >60% of that in NAb-free controls across multiple IVIG dilutions (Figure 4B) and inhibited mNAbs more effectively than the control peptides (Figure 4C).

To test patient variability, plasma was collected from 20 individuals with hemophilia A or B. NAb titers varied widely, with nine patients showing pre-existing titers >1:64 (Figures 4D and 4E). Preincubation with the decoy mixture reduced NAb titers below 1:8 in six of these nine patients (Figure 4F), demonstrating its ability to reduce NAb activity in samples with clinically relevant titers. In plasma from BBM901-treated patients, in which titers exceeded 1:100,000, we tested a dual strategy combining IdeS with decoy peptide treatment. IdeS alone reduced titers to ∼1:1,000, while subsequent peptide adsorption lowered residual titers further to ∼1:4 (Figures 4G and 4H). These findings demonstrate that peptide mixtures, particularly when combined with IdeS, can overcome even extreme NAb levels *in vitro*, modeling an AAV re-administration setting.

To assess whether decoy peptides interfere with receptor-mediated AAV entry, Huh7 cells were pre-incubated with peptide mixtures at low, medium, and high concentrations before AAV infection. No significant differences in transduction efficiency were observed compared to PBS-treated controls (Figures 4I and 4J), indicating that peptide treatment does not impair receptor-mediated viral entry.

### Decoy peptide mixtures restore gene therapy efficacy in pre-existing and repeated dosing NAb models

**Figure 5:**
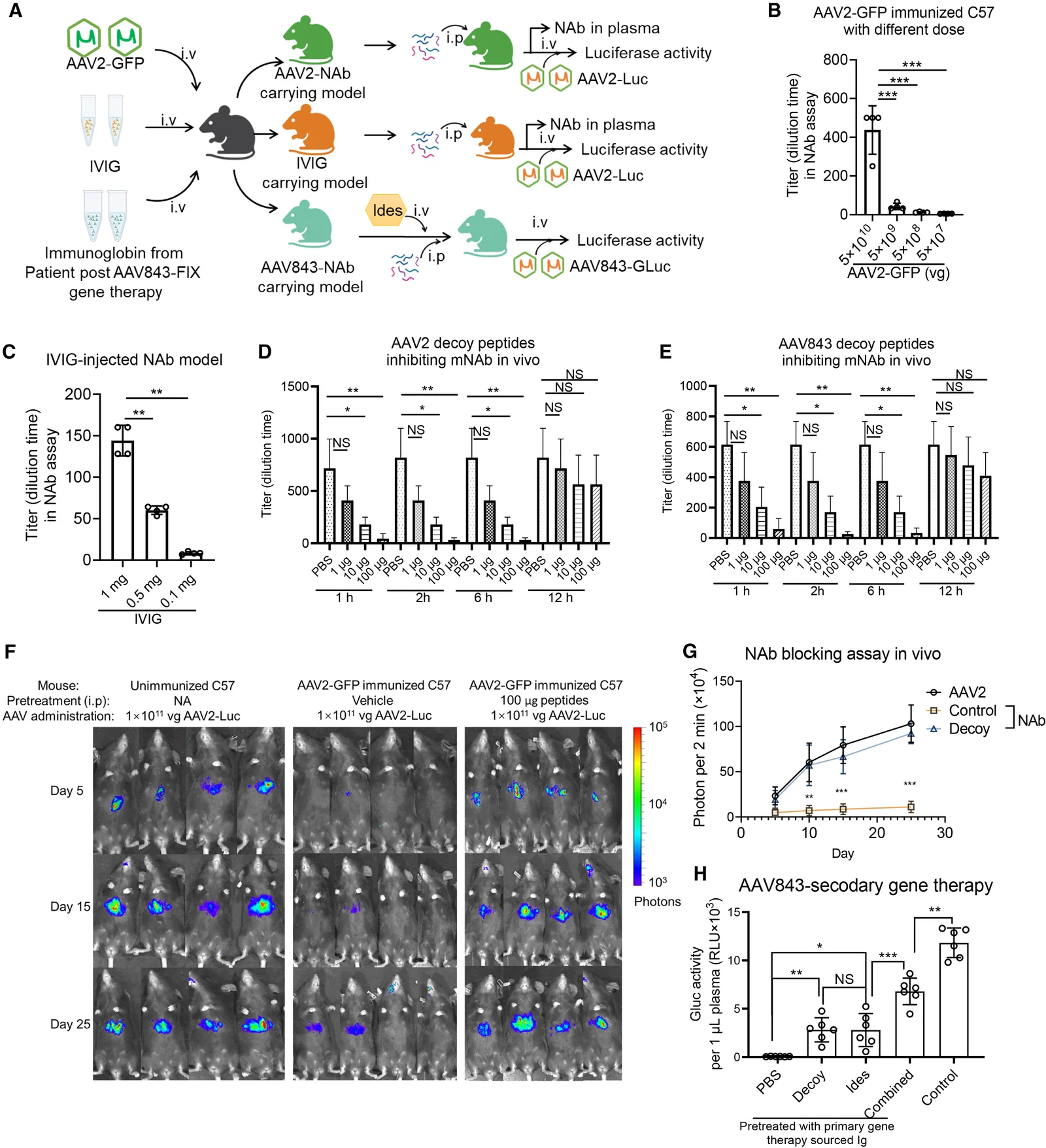
Decoy peptide mixtures suppress NAbs and restore AAV gene therapy efficacy in pre-existing and repeated dosing models (A) Schematic of *in vivo* models: IVIG-pre-existing NAbs, repeated dosing after AAV2 immunization, and high-titer NAbs using patient-derived Ig. (B and C) Dose-response of NAb titers after injection of AAV2-GFP (5 × 10^7^–5 × 10^10^ vector genomes (vg), i.v.) or IVIG (0.1–1 mg, i.v.) in C57BL/6 mice (*n* = 4 per group). Plasma was collected at day 14 post-AAV injection or day 1 post-IVIG administration and tested by NAb assay. (D and E) *In vivo* kinetics of peptide-mediated NAb inhibition. C57BL/6 mice were first immunized with AAV vectors (5 × 10^10^ vg), and NAb titers were detected at day 21. Mice were then administered decoy peptide mixtures (i.p.), and plasma samples were collected at the indicated time points and NAb titer was measured. (F and G) C57BL/6 mice were first immunized with AAV vectors (5 × 10^10^ vg per mouse) for 14 days to establish NAb, followed by a second administration of AAV2-Luc (1 × 10^11^ vg per mouse). Prior to re-dosing, mice received decoy peptide mixtures (total dose: 10 μg per mouse, i.p.). Luciferase expression in the liver was monitored by *in vivo* bioluminescence imaging (IVIS) at days 5–25 after the second AAV administration. (H) Human NAb model established with patient-derived Ig (containing AAV843 Nab, 1 mg/mouse, i.v.) at 0 h. Combination of IdeS (1,000 U, i.v. at 1 h) and AAV843-decoys (10 μg per mouse, i.p. at 12 h) enabled redosing with AAV843-Gluc (5 × 10^11^ vg, i.v. at 16 h). Plasma Gluc activity was measured 4 weeks later.

To evaluate therapeutic efficacy *in vivo*, we established three mouse models (Figure 5A): an IVIG pre-existing NAb model by intravenous (i.v.) injection of human IVIG; a repeated dosing model by active immunization with AAV2 particles; and a high-titer NAb model using patient-derived immunoglobulins purified from BBM901-treated hemophilia B patients.

Dose-response studies confirmed that increasing amounts of IVIG (0.1–1 mg) or AAV2-GFP particles (5 × 10^7^–5 × 10^10^ vg) produced proportionally higher NAb titers by day 1 or day 14, respectively (Figures 5B and 5C). These results established baseline dose-NAb relationships for subsequent inhibition studies.

To assess the *in vivo* duration of peptide-mediated NAb inhibition, C57BL/6 mice immunized with 5 × 10^10^ vg of AAV (NAb titers 1:512–1:2,048) were treated with peptide mixtures and plasma samples were collected over time. A rapid reduction in NAb titers was observed within 1 h, reaching levels of approximately 1:32–1:64. This inhibitory effect was maintained for at least 6 h (Figures 5D and 5E). These findings suggest that although free peptides may have short half-lives, the peptides provided sustained functional NAb inhibition, creating a therapeutic window for AAV re-administration.

When mice were pretreated with decoys before AAV2-Luc infusion, liver Luc expression was significantly restored in both IVIG-pretreated and AAV2-immunized models, reaching levels comparable to NAb-free controls (Figures 5F, 5G, and S10).

Finally, in the third model (mimicking clinical redosing), IgG was purified using Protein A/G from the plasma of BBM901-treated patients with hemophilia B (AAV843 NAb titers >1:10,000) and administered to mice to establish a high-titer NAb model. Here, combined administration of IdeS (i.v.) followed by AAV843-derived decoy peptides (intraperitoneal, i.p.) enabled successful re-administration of AAV843-Gaussia Luc (Gluc). Four weeks later, plasma Gluc activity reached ∼60% of the NAb-free control and showed a >1,000-fold increase over untreated mice (Figure 5H).

Together, these results demonstrate that decoy peptides can suppress NAbs sufficiently to restore AAV transduction *in vivo*, both in pre-existing and repeated dosing settings, and that combining IdeS with decoys is effective in overcoming even extreme antibody titers.

### Decoy peptides exhibited weak predicted MHC class II binding affinity and did not elicit detectable immune responses following i.p. administration *in vivo*

**Figure 6:**
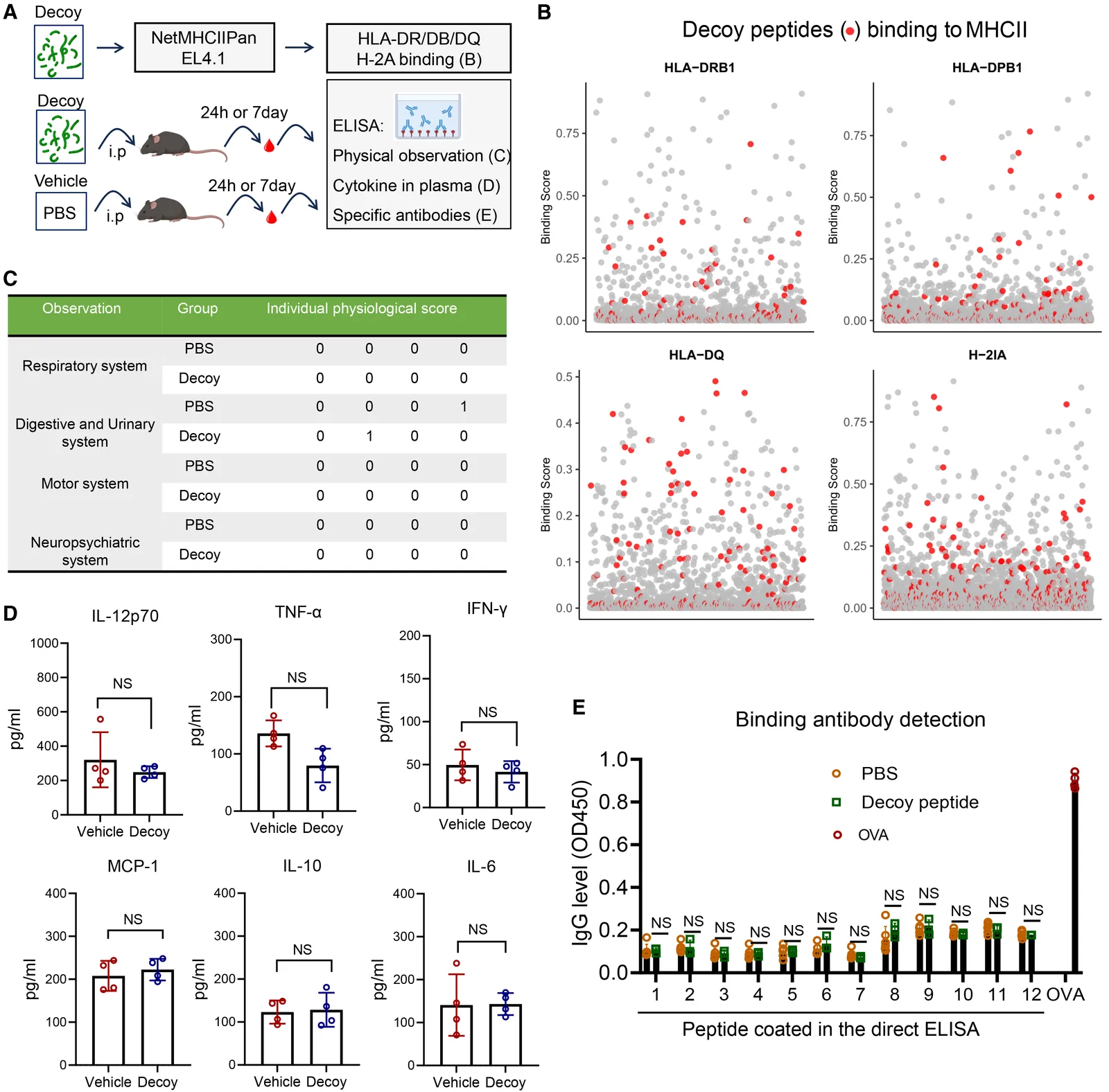
Safety evaluation of AAV2 decoy peptides by MHC-II binding prediction and *in vivo* testing (A) Workflow for intraperitoneal administration of peptide mixtures in mice to evaluate potential safety risks, including systemic physiology, cytokine responses, and antibody induction. (B) Predicted MHC-II binding affinity of overlapping 12–15 mer peptides spanning the AAV2 capsid sequence using the IEDB platform, with decoy peptide regions indicated. (C) Physiological monitoring of mice following high-dose peptide administration, including neurological, metabolic, motor, and hematologic parameters. (D) Plasma cytokine concentrations (IL-12p70, TNF-α, IFN-γ, MCP-1, IL-10, and IL-6) measured 24 h post-injection. (E) Detection of peptide-specific IgG in plasma by ELISA following peptide or control antigen administration. Numbers on the *x* axis represent individual AAV2 decoy peptide identifiers, corresponding to Table S2.

While individual antibody repertoires may vary, the use of multi-epitope peptide pools enables broader coverage of neutralizing antibody specificities. If peptide mixtures are to be applied clinically, ensuring the safety of the product is of paramount concern. As shown in Figure 6A, we performed an *in vivo* i.p. peptide administration experiment to detect antibody responses, inflammatory factors, and physiological reactions induced by peptide mixture injection. Before *in vivo* testing, we first evaluated potential immunogenicity using the Immune Epitope Database (IEDB) MHC-II binding prediction tool. Overlapping 12–15 mer peptides spanning the AAV2 capsid were scored against common human and murine MHC-II alleles. The predicted epitopes corresponding to the AAV2 decoy peptides (highlighted in red, Figure 6B) consistently showed weak binding affinity, indicating limited predicted MHC-II binding. In both our gene therapy models and experiments involving a single injection of a high-dose peptide mixture, we observed no alterations in neurological, metabolic, motor, or hematologic parameters within 1 week (Figure 6C). No increases in the measured cytokines were detected (Figure 6D), providing preliminary evidence for the safety of the peptide mixture. To assess potential immunogenicity following peptide administration, we performed enzyme-linked immunosorbent assays (ELISA) to measure peptide-specific IgG responses. No increase in peptide-specific IgG levels was detected in plasma at 1 week post-injection, whereas the OVA positive control elicited a robust antibody response under the same conditions (Figure 6E).

These findings suggest that i.p. administration of the peptide mixture does not induce detectable early cytokine or peptide-specific IgG responses at this early time point, supporting its preliminary safety profile.

### AI-generated peptides expand predicted epitope coverage and inhibit NAbs

**Figure 7:**
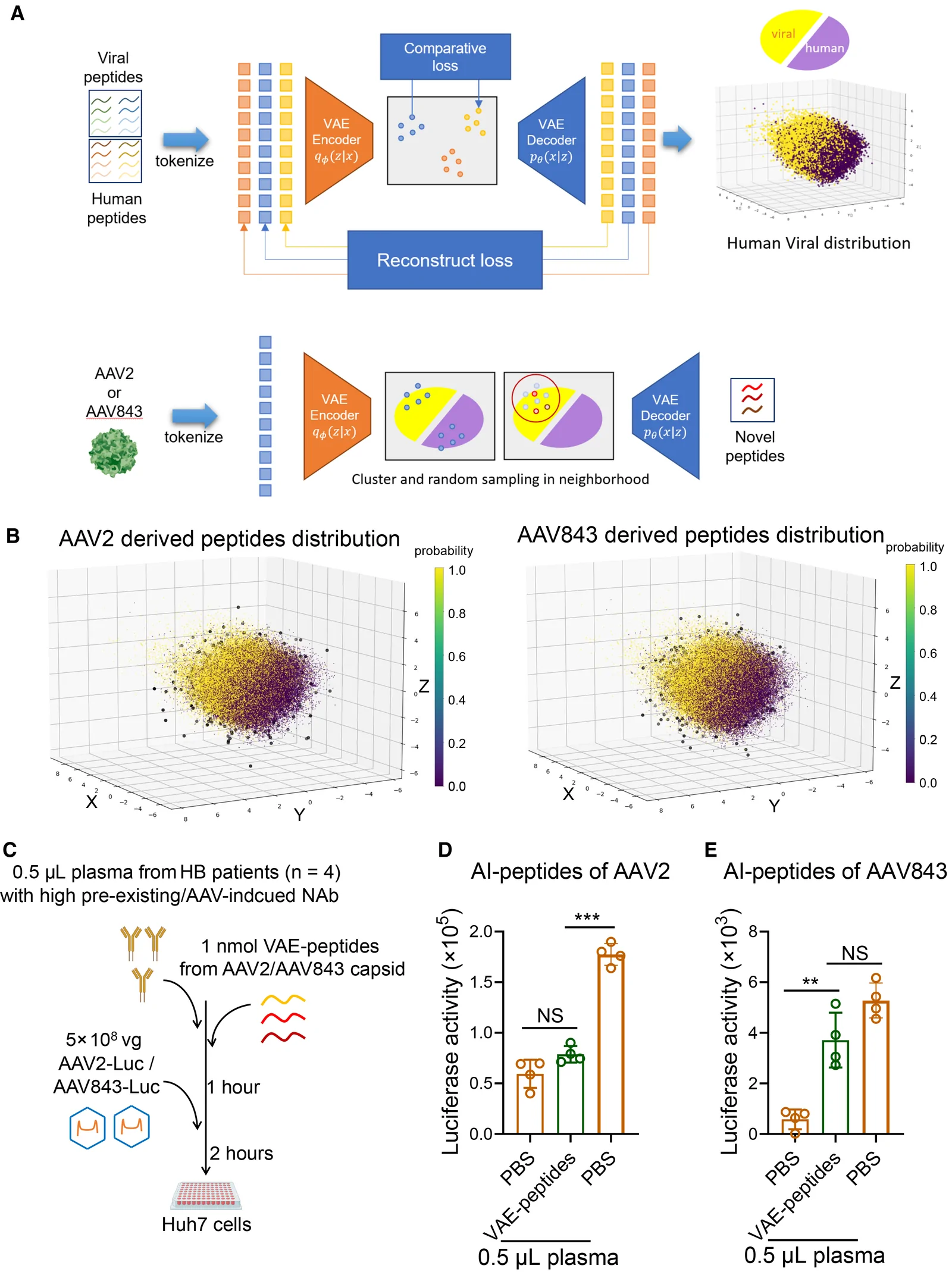
Generation of AI-derived non-native peptides using the VAE model and validation of NAb inhibition (A) Schematic representation of the VAE model framework and the process of generating non-native peptides. (B) Distribution of AAV2- and AAV843-derived peptides within the VAE latent space. (C–E) Experimental validation of the NAb-inhibitory activity of VAE-derived decoy peptides. (C) Workflow illustrating the validation process. (D and E) Comparative analysis of NAb inhibition by VAE-derived peptides, assessed through luciferase activity in the NAb assay.

To explore whether AI could generate novel inhibitory sequences beyond naturally derived capsid peptides, we leveraged the generative capacity of the pretrained LoRA-adapted ESM2 model. A VAE was developed in which the encoder compressed input sequences into latent variables and the decoder reconstructed amino acid sequences. To refine peptide discrimination, we integrated a contrastive learning loss that minimized distances between human-derived peptides while maximizing divergence from pathogen-derived peptides, to improve discrimination between host- and virus-derived sequence features (Figure 7A).

During sequence generation, the model extracted sequence features of AAV capsid proteins in the latent space. By combining k-means clustering with Monte Carlo sampling, the VAE produced novel decoy peptides that were distinct from naturally occurring capsid-derived peptides (Figure 7B; Table S3).

We next tested these AI-generated peptides in functional inhibition assays (Figure 7C). When incubated with plasma from hemophilia B patients carrying high-titer AAV2 NAbs (>1:256), VAE-generated peptide candidates for AAV2 showed minimal inhibition (Figure 7D). In contrast, VAE-generated peptide candidates for AAV843 significantly reduced NAb-mediated inhibition in patient plasma with extremely high NAb titers (>1:10,000 after BBM901 therapy), restoring measurable transduction levels (Figure 7E).

Together, these findings show that AI-generated non-native peptides can expand epitope coverage and neutralize high-titer NAbs. This highlights the potential of generative modeling to complement epitope prediction and design inhibitory peptides not present in natural capsid sequences, thereby broadening the therapeutic toolbox for AAV gene therapy.

## Discussion

Our findings support a decoy mechanism based on pre-occupancy of NAbs, in which peptides engage circulating antibodies prior to viral exposure. This pre-exposure sequestration represents a temporally separated form of competitive inhibition and enables effective functional blockade of NAbs despite the lower apparent binding affinity of individual peptides compared to intact AAV capsids. This approach differs in format from empty capsid decoys and vector shielding strategies, which rely on structural mimicry or physical blocking.

In this context, we demonstrate that sequence-based AI frameworks, including Transformer-based models such as BERT and ESM-LoRA, can effectively identify and prioritize AAV capsid-derived peptide candidates for antibody modulation. By integrating hydrophilicity filtering with polarity-based classification, the pipeline reduced the experimental search space from over 1,400 peptides to 39 candidates (Table S2). These models consistently achieved F1 scores above 0.80 and outperformed classical sequence-based classifiers (e.g., Random Forest and NLP-Metaxa), while also exceeding the performance of structure-based patch prediction approaches such as MaSIF,^28^ which showed lower accuracy (<0.80; Figure S11). Importantly, rather than serving as a direct predictor of epitopes, this framework provides a sequence-level prioritization strategy that can be coupled with structural mapping and functional validation. This sequence-level prioritization strategy may enable rapid and scalable identification of candidate decoys across diverse AAV serotypes, particularly in settings where structural data are incomplete.

Functional assays further confirmed that the selected decoy peptides preferentially bind AAV-targeted NAbs and effectively inhibit antibody-mediated neutralization. In both *in vitro* and *in vivo* models, peptide treatment restored AAV transduction, with approximately 60% recovery of reporter gene expression observed in murine systems under NAb challenge. Moreover, combination treatment with IdeS followed by decoy peptide administration markedly reduced NAb titers from >1:10,000 to approximately 1:4, demonstrating synergistic effects. This result is consistent with, yet mechanistically distinct from, previously reported capsid-based decoy strategies,^29^ as peptide-mediated sequestration does not require intact viral particles, multivalent capsid structures, or high-avidity interactions typically associated with capsid-based decoys.

The prevalence of pre-existing NAbs remains a major limitation for AAV gene therapy, with approximately 50% of individuals harboring antibodies against common serotypes such as AAV2-AAV5 at clinically relevant levels.^30^ Current strategies, including empty capsid decoys,^13^ plasmapheresis,^31^ and immunosuppressive regimens,^31^ either lack specificity or carry substantial clinical risks. In contrast, the peptide-based strategy described here provides a mechanistically distinct, targeted, and modular alternative that operates through antibody engagement rather than antibody depletion or vector shielding. Consistent with this, safety evaluation in mice, including cytokine profiling, behavioral assessment, MHC-II binding prediction, and peptide-specific antibody monitoring, revealed no detectable immunotoxicity or peptide immunogenicity even at high doses. This distinction may be particularly advantageous in clinical contexts where antibody levels are difficult to fully eliminate or where preservation of vector functionality is critical.

Despite these promising findings, several limitations should be considered. First, although short peptides typically exhibit rapid clearance *in vivo*, our data suggest that antibody-bound peptide complexes may extend functional activity, creating a transient therapeutic window for AAV administration. Second, variability in antibody repertoires across individuals may influence the efficacy of a fixed peptide mixture, particularly in high-titer settings. Indeed, while the combination of IdeS and peptides was effective even in models using patient-derived high-titer IgG (>1:10,000), this model may overestimate the accessibility of circulating antibodies compared to physiological conditions, and thus may allow partial transduction even under high nominal NAb titers. Third, while the VAE-derived peptides showed promising activity against AAV843-directed NAbs, their performance against AAV2 suggests that generative model refinement and integration of structural information may further improve targeting precision. In addition, key parameters such as baseline NAb titers, vector dose, route of administration, and target tissue may further influence the efficacy of this approach and warrant systematic evaluation.

Finally, although the current study demonstrates efficacy in murine systems, translation to clinical application will require validation in nonhuman primates to assess pharmacokinetics, immunological safety, and durability of response.

In summary, this study establishes a sequence-guided, interpretable, and scalable peptide decoy platform for modulating antibody activity. By addressing both pre-existing and therapy-induced NAbs, this approach may expand patient eligibility and enable repeat dosing in AAV-based gene therapy, with potential applicability to other antibody-mediated therapeutic contexts.

## Materials and methods

### The acquisition of clinical specimens and ethical considerations

Twenty plasma samples were obtained from patients diagnosed with hemophilia A or B who had not received AAV gene therapy, and peripheral blood plasma was collected from five patients with hemophilia B at 26 weeks after AAV-based gene therapy. Written informed consent was obtained from all participants prior to sample collection. All procedures involving human participants were approved by the Ethics Committee of the Institute of Hematology and Blood Diseases, Chinese Academy of Medical Sciences (approval no. CAMSCRF2021013-EC-2) and conducted in accordance with the principles of the Declaration of Helsinki. All animal experiments were approved by the Ethics Committee of the Institute of Hematology and Blood Diseases, Chinese Academy of Medical Sciences (approval no. IHCAMS-DWLL-CIFMS2025064-2), and were conducted in accordance with institutional guidelines and relevant national regulations.

### Construction of human and viral amino acid sequence libraries

Initially, protein names and amino acid sequences were obtained from the Uniprot-KB database and categorized by taxonomy. The viral dataset included proteins from diverse viral families and was not restricted to AAV, allowing the model to learn generalizable virus-specific sequence features. Only secreted or membrane proteins of human origin were chosen. Peptide sequences ranging from 5- to 50-mer were derived using a one-residue step sliding window method. The GRAVY algorithm^32^ was employed to assess the hydrophobicity of these peptides, retaining those with GRAVY scores below −1.0 to establish class-specific peptide libraries. To reduce redundancy, CD-HIT^33^ (v.4.8.1) clustering was applied with a 30% sequence identity threshold. Sequences were clustered at this threshold, with one representative retained from each cluster. Following this, 300,000 peptide sequences were randomly sampled from each species-specific library. These sequences were proportionally divided, with 250,000 sequences allocated to the training set and 50,000 sequences to the validation set in each group. The database was generated on a server with Ubuntu 18.04, CPU model Intel (R) Xeon (R) Gold 6258R, and GPU model NVIDIA GeForce RTX5090.

### Embedding and input representation

Transformer-based models, BERT and ESM2 with LoRA, were used to encode raw protein sequences into contextual embeddings. Amino acids were tokenized and converted into dense vectors with positional encodings, preserving residue identity and sequential order. These contextualized representations capture position-dependent biochemical patterns essential for downstream peptide classification and generation tasks.

### Model architectures

#### BERT

We employed a domain-specific BERT architecture, adopting the original Transformer encoder framework. Each layer comprises a multi-head self-attention (MHSA) module followed by a position-wise feedforward network (FFN), each with residual connections and layer normalization. The model processes sequences of up to 1,024 aa, with each residue embedded into a 768-dimensional vector, forming a sequence matrix $H^{l}\in R^{L\times d}$, where *L* ≤ 1024 denotes the sequence length and, *d* = 768 is the hidden (embedding) dimension.

At layer *l*, the hidden representation $H^{l}\in R^{L\times d}$ is updated by sequentially applying the MHSA and FFN modules to the input states, each followed by a residual connection and layer normalization:

(Equation 1) $$H^{l+1}=\text{LayerNorm}(H^{l}+\text{FFN}\left(\text{MHSA}\left(H^{l}\right)\right)$$

Three reduced-depth configurations were used: 1, 2, and 4 layers, each with 16 attention heads, hidden size 768, and FFN dimension 3,072. Pretraining used the masked language modeling (MLM) objective to capture contextual residue semantics.

#### ESM2 with LoRA

The ESM2 model (facebook/esm2_t33_650M_UR50D) extends the Transformer encoder to a deeper and wider architecture, featuring 33 layers, each with 16 attention heads, a hidden size of 1,280, and an FFN dimension of 4,096.

To adapt ESM2 to domain-specific protein datasets, we applied LoRA to the attention modules during post-training. LoRA introduces trainable low-rank matrices into the query (Q), key (K), and value (V) projection layers. This allows efficient fine-tuning while freezing most of the pretrained parameters.

Formally, for a pretrained projection matrix *W*, LoRA reparameterizes it as:

(Equation 2) $$\tilde{W_{p}^{\left(l\right)}}=W_{p}^{\left(l\right)}+\alpha \cdot A_{p}^{\left(l\right)}B_{p}^{\left(l\right)},\;P\in \{Q,K,V\}$$

where $B_{p}^{l}\in {\mathbb{R}}^{d\times r},A_{p}^{l}\in {\mathbb{R}}^{r\times k},r\ll \min\left(d,k\right)$, and *α* is a scale factor. This parameter-efficient mechanism retains generalization ability while enabling task-specific adaptation.

#### Transformer components

Both BERT and ESM2 share the same underlying Transformer architecture, consisting of the following core components.

#### MHSA

At each layer, the input hidden states $H\in R^{L\times d}$ are linearly projected into query *Q*, key *K*, and value *V* matrices using learned weight matrices $W_{Q,i}^{\left(l\right)},W_{K,i}^{\left(l\right)},W_{V,i}^{\left(l\right)}\in {\mathbb{R}}^{d\times d_{k}}$, *i* represents the *i*-th head of multi head attention, and *l* represents the output of the *l*-th layer :

(Equation 3) $$Q_{i}^{\left(l\right)}=H^{l}W_{Q,i}^{\left(l\right)},K_{i}^{\left(l\right)}=H^{l}W_{K,i}^{\left(l\right)},V_{i}^{\left(l\right)}=H^{l}W_{V,i}^{\left(l\right)}$$

The attention output for each head is computed as:

(Equation 4) $$\text{Attention}\left(Q_{i}^{\left(l\right)},K_{i}^{\left(l\right)},V_{i}^{\left(l\right)}\right)=\text{softmax}\left(\frac{Q_{i}^{\left(l\right)}{K_{i}^{\left(l\right)}}^{T}}{\sqrt{d_{k}}}\right)V_{i}^{\left(l\right)}$$

Multi-head attention combines multiple parallel attention mechanisms:

(Equation 5) $${\text{MHSA}}^{\left(l\right)}\left(H^{l}\right)=\text{Concat}\left(head_{1}^{\left(l\right)},head_{2}^{\left(l\right)},\ldots ,head_{n}^{\left(l\right)}\right)W_{O}^{\left(l\right)}$$

where each head is computed as:

(Equation 6) $$head_{i}^{\left(l\right)}=\text{Attention}\left(Q_{i}^{\left(l\right)},K_{i}^{\left(l\right)},V_{i}^{\left(l\right)}\right)$$

#### Position-wise FFN

In both BERT and ESM2 models, each Transformer layer contains a position-wise FFN that processes the output of the multi-head attention mechanism.

(Equation 7) $$\text{FFN}\left(x\right)=\text{ReLU}\left(0,xW_{1}+b_{1}\right)W_{2}+b_{2}$$

where $W_{1}\in R^{d\times s},W_{2}\in R^{s\times d}$ are weight matrices for the first and second layers. $b_{1}\in R^{L\times s},b_{2}\in R^{L\times d}$ are bias terms. Here, *s* is the dimension of FFN. And Rectified Linear Unit (ReLU) is the activation function ReLU(*x*) = *max*{0,*x*}, which allows the model to apply nonlinear transformations to each token embedding.

Layer normalization and residual connections: after each attention and FFN block, residual connections are followed by layer normalization, stabilizing training, and accelerating convergence.

(Equation 8) Residualconnection(x)=x+FFN(x)

(Equation 9) $$\text{Layer}\;\text{norm}\left(x\right)=\frac{x-\mu }{\sigma }\cdot \gamma +\beta$$

(Equation 10) $$\mu =\frac{1}{d}\sum_{i=1}^{d}x_{i},\sigma =\sqrt{\frac{1}{d}{\sum_{i}^{d}\left(x_{i}-\mu \right)}^{2}}$$

Where Equation 10 is the mean and standard deviation of input feature $x\in R^{d}$ respectively, *γ* and *β* are learnable affine parameters.

#### Pretraining phase

The training pipeline for both the BERT-based model and the ESM2 with LoRA model involves a combination of unsupervised pre-training or post-training, followed by supervised fine-tuning for classification.

#### BERT pre-training and fine-tuning

The BERT-based model follows an unsupervised pre-training paradigm using the MLM objective. During pre-training, 30% of tokens were randomly masked. This masking ratio is higher than that used in natural language BERT models (typically 15%) but has been shown to be effective for protein sequence modeling,^25^ where higher masking ratios can improve contextual representation learning due to the redundancy and local dependencies in amino acid sequences. The model is then trained to reconstruct the original tokens based solely on their context. The MLM loss measures the negative log likelihood of correctly predicting the original identity of masked tokens based on the surrounding context. We use the AdamW optimizer^34^ with initial learning rate 0.00003 for training, The total training epochs is 30 and the batch size is 16.

After pretraining, we fine-tune the model on a downstream protein classification task using labeled data. Fine-tuning involves optimizing the model to predict class labels for peptide sequences.

To perform classification, a special [CLS] token was prepended to each input sequence. This token is designed to aggregate global information across the entire sequence. After the input passes through all Transformer layers, we extract the final hidden representation of the [CLS] token. This vector is then passed through a linear classification head followed by a softmax function to produce class probabilities. The training objective is the standard cross-entropy loss between predicted probabilities and ground-truth labels.

We fine-tune the model for 30 epochs with a batch size of 256, using the AdamW optimizer with an initial learning rate of 2e − 5. A cosine learning rate schedule is applied with a warm-up ratio of 0.01.

#### ESM-LoRA post-training and fine-tuning

The ESM-LoRA model follows the same unsupervised pre-training paradigm as the BERT-based model, using the MLM objective during post-training and the same cross-entropy loss during supervised fine-tuning.

In our configuration, the LoRA rank both in post-training and fine-tuning steps *r* is set to 16, the scaling factor *α* to 32, and a dropout rate of 0.1 is applied during training. The model is optimized using the AdamW optimizer with a per-device batch size of 2 during the post-training stage and 32 during fine-tuning. It is trained for 10 epochs using a learning rate of 5e − 5 and weight decay of 0.01.

By incorporating LoRA into the ESM post-training phase, we enabled the model to adapt more effectively to task-specific distributions while substantially reducing the number of trainable parameters relative to full-model fine-tuning.

#### 3D structure analysis of AAV capsid

The AAV2 protein conformational structure, assembled from PDB entry “6ih9,” commences at amino acid residue 219 of the VP3 protein sequence. And the AAV843 VP3 conformational structure was predicted by AlphaFold3 according to the amino acid sequence of VP3. Utilizing Chimera-X software (v.1.70), the PDB file containing the protein conformational structure is accessed, with amino acid sequences color-coded in accordance with the software’s guidelines.

#### Viral score calculation

For AAV capsid analysis, full-length VP1 amino acid sequences were used as input. Sliding window segmentation (20-mer, step size = 1) was applied directly to the protein sequences. As VP2 and VP3 represent N-terminally truncated forms of VP1, this approach inherently captures all peptide regions present in the mature capsid proteins. All analyses were performed at the amino acid level, independent of nucleotide reading frames. These sequences underwent classification prediction using the BERT model. Peptides were predicted to be in the viral group or human group by probability. Subsequently, we computed a viral score for the core position of each peptide. For instance, in the AAV2-derived peptides, the core position of the first peptide (1–20 mer) was defined as position 10, while the core position of the second peptide was defined as position 11, and so forth. The viral score for each core position was calculated as the cumulative sum of the viral probabilities of its corresponding peptide and the preceding nine and following ten peptides, as defined by the formula subsequently. A higher viral score indicates a higher local cumulative viral-class probability.

(Equation 11) $$Viral\;Score\left(x_{i}\right)=\sum_{j\in N_{10}\left(x_{i}\right)}\frac{model\left(x_{j}\right)}{\left\|N_{10}\left(x_{i}\right)\right\|}$$

(Equation 12) $$N_{\epsilon }\left(x_{i}\right)=\{j|j\in \left[i-\epsilon ,i+\epsilon \right]\}$$

(Equation 13) $$\left\|N_{\epsilon }\left(x_{i}\right)\right\|=\min\left(i+\epsilon ,L\right)-\max\left(i-\epsilon \right)$$

#### VAE model construction and intelligent decoy peptide generation

During model development, we constructed a VAE framework based on post-trained ESM-2 protein language models to investigate the reconstruction and representation of 20-residue peptide sequences composed of 20 canonical amino acids. The VAE is a type of generative neural network that learns to encode high-dimensional biological sequences into a structured, lower-dimensional latent space, and to reconstruct the original sequences from this compressed representation. Specifically, the encoder maps input peptide sequences *x* into continuous latent variables (feature in Figure 1A) *z* = encoder(*x*), capturing their essential biological features, while the decoder attempts to regenerate the original sequences from these latent codes $\hat{x}=\text{decoder}\left(x\right)$. This allows the model to learn underlying patterns and constraints of peptide sequences in an unsupervised manner.

To enhance the model’s ability to distinguish host-specific features, we introduced an innovative contrastive learning mechanism. We employed the pre-trained ESM-2 model as the encoder backbone, excluding its final feedforward classification head to retain only its feature extraction capability. By designing a dual-modal loss function, the classification head was trained in parallel for a supervised classification task, allowing the model to simultaneously optimize for both sequence reconstruction and downstream discrimination of peptide origin:

(Equation 14) $$L_{dual}\left(x_{i},{\hat{x}}_{i},y,{\hat{y}}_{i}\right)=L_{CE}\left(y,{\hat{y}}_{i}\right)+L_{CL}\left(x_{i},{\hat{x}}_{i},y,{\hat{y}}_{i}\right)$$

(Equation 15) $$L_{CL}\left(x_{i},{\hat{x}}_{i},y,{\hat{y}}_{i}\right)=\frac{1}{N^{2}}\sum_{i=1}^{N}\sum_{j=1}^{N}\left[S_{ij}\cdot D_{ij}^{2}+\left(1-S_{ij}\right)\cdot {\left(\max\left(0,m-D_{ij}\right)\right)}^{2}\right]$$

where *y*_*i*_ is the class label for sample *i*, and ${\hat{y}}_{i}$ is the prediction by the ESM model classification head. $D_{ij}={\left\|z_{i}-z_{j}\right\|}_{2}$ is the Euclidean distance between the encoded features of two different samples *i*,*j*. *S*_*ij*_ encourages embeddings from the same class to be close, while (1−*S*_*ij*_) penalizes embeddings from different classes when they are too close. *m* is a margin hyperparameter to control the minimum allowable distance between embeddings of different classes in the feature space.

This approach minimizes the latent variable distance between human-derived peptides while maximizing their feature divergence from virus-derived peptides. This contrastive learning strategy enables the trained VAE to establish distinct boundaries in latent space, thereby providing reliable embedded representations for accurate discrimination between human-derived and virus-derived peptides.

In the peptide generation phase, we proposed an intelligent decoy peptide generation strategy based on the hypothesis of a positive correlation between host classification and immunogenicity characterization. Following the principle of latent space proximity (where geometric distance between latent variables correlates with immunogenic similarity), the pre-trained encoder was used to extract deep features from 20-mer sequences derived from AAV capsid proteins. Subsequently, the k-means unsupervised clustering algorithm was applied to construct feature clusters in the low-dimensional latent space. Finally, importance sampling was performed in the central region of the target clusters using the Monte Carlo method, and the sampled latent variables were transformed into novel artificial decoy peptides with predefined sequence-classification properties through a fine-tuned decoder.

#### The short decoy peptides were prioritized over 1,400 peptide candidates

To identify optimal inhibitory candidates, we compared peptide lengths for distinguishing human-from virus-derived sequences using the fine-tuned BERT classifier. The highest discrimination accuracy was observed for 20-mers, which were thus selected as the screening unit. Using a one–amino acid sliding window, all overlapping 20-mer peptides from the VP1 capsids of AAV2 (735 aa) and AAV843 (737 aa) were generated, yielding 1,434 candidates. Each sequence was scored by the BERT model for viral versus human probability, and a cumulative-sum smoothing algorithm was applied to reduce local fluctuations. Peptides were filtered by (1) viral-classification probability threshold and (2) confirmed capsid surface exposure. Fragments shorter than 20 aa were symmetrically extended, and longer regions were segmented with 5–15 aa overlaps. Ultimately, 39 AAV2- or AAV843-derived 20-mer peptides were synthesized (>95% purity) and validated experimentally.

#### Peptide synthesis

The peptides utilized in this investigation were synthesized by Nanjing Peptide Corporation. Following synthesis, peptides underwent purification via high-performance liquid chromatography to achieve a purity exceeding 95%. Subsequently, peptide powder was dissolved in PBS or DMSO to create a storage solution with a concentration of 1 mM, which was then stored at −80°C for a maximum duration of 3 months. Prior to subsequent experiments, peptides were diluted in PBS. No carrier proteins (e.g., BSA) were used during peptide preparation or dilution.

#### Packaging and quantification of AAV virus and empty capsids lacking packaged genomes

AAV viral and empty capsids were produced by triple- or double-plasmid transfection (pAAV, pREP/CAP, pXX680^35^) in HEK293 cells using Polyethylenimine (PEI) complexation. Viral particles were harvested from both supernatant and cell lysates, purified by iodixanol gradient ultracentrifugation, and verified by SDS-PAGE and Coomassie staining. Quantification of full AAV particles (genome-containing AAV) was performed by qPCR based on vg titers. For empty capsids, equivalent amounts were estimated based on capsid protein levels using SDS-PAGE followed by Coomassie staining. Band intensities of VP1, VP2, and VP3 were compared with those of full particles with known vg titers to approximate capsid-equivalent concentrations. Promoters used were CMV (AAV2-GFP, AAV2-Luc) and TTR (AAV843-GLuc).

#### Quantification of AAV particles and biotin-labeled peptides

AAV2 purification and peptide synthesis, purification, and Quality Control followed established protocols.^36^ The initial AAV2 titer was 2 × 10^9^ vg/μL (∼200 nM antigen equivalent), and peptides were prepared at 2 mg/mL (∼800 μM, ∼2.5 kDa). Biotinylation was performed using the Biotin-NHS kit (Thermo Fisher Scientific #20217) per manufacturer instructions: 0.1 mg peptide or 1 × 10^11^ vg AAV2 in a 50 μL reaction. After labeling, biotin-AAV2 was purified using Amicon UFC5100 ultrafiltration columns, while biotin-peptides were desalted with Pierce 89852 columns. Both products were re-quantified using the Pierce 23227 protein assay.

#### *In vitro* NAbs assay

A standardized NAb assay was performed to ensure detection stability and reproducibility. Mouse plasma (4 μL) was serially diluted 2-fold starting from 1:2 in DMEM (Gibco #11965092) across 12 gradients, yielding a maximum dilution of 1:4,096. AAV2-Luc (4 × 10^8^ vg) was added to each dilution and incubated at 37°C for 30 min, then mixed with 2 × 10^5^ HEK293 cells (MOI = 2,000). After 48 h, Luc activity was measured using a Promega E1500 kit. NAb titers were defined as the highest plasma dilution resulting in ≥50% inhibition of transduction. All samples were tested in triplicate.

For blocking assays, peptides or empty capsids (normalized to AAV vg equivalents) were preincubated with diluted plasma for 1 h at 37°C prior to AAV2-Luc addition, followed by the same workflow described above. The input plasma volume (4 μL) was sufficient to support serial dilution and replicate measurements within the assay format.

#### Purification and conjugation of antibodies to latex beads

Human IVIG was sourced from Chengdu Rongsheng Pharmaceuticals at a concentration of 2.5 mg/mL. The mNAbs were harvested from female C57 mice aged 6–8 weeks, 2 weeks following the i.v. administration of 1 × 10^11^ vg of AAV2. Anesthesia with isoflurane was employed, and blood was collected via retro-orbital sampling (approximately 200 μL per mouse from five mice, pooled to obtain a total of 1 mL). The antibodies within the mouse plasma were adsorbed and purified using Protein A/G columns (Thermo Fisher Scientific #89958). Subsequently, solvent exchange was performed, and the antibodies were concentrated to 1 mg/mL utilizing ultrafiltration tubes (Amicon #UFC803008). A specific quantity of either IVIG or mNAbs was incubated with latex beads (Invitrogen #A37306) to facilitate the conjugation of antibodies with the beads. The concentration of beads was determined using flow cytometry to ensure that each 1 μL of beads contained over 100,000 individual bead particles.

#### Affinity analysis of AAV particles and peptides binding to specific antibodies via saturation or competitive binding

In the saturation binding experiment, biotin-AAV2 or AAV843 particles or biotin-peptides at varying concentrations (0–8,000 nM) were incubated with 1 μL latex beads conjugated with IVIG, mNAbs, or NAbs in 20 μL PBS at room temperature for 30 min. Following centrifugation at 500× g for 5 min and removal of the supernatant, the beads were resuspended in 20 μL PBS containing 0.25 μg PE-Streptavidin (BD #349023) and incubated in the dark for an additional 30 min. Subsequently, 50 μL PBS was added, and analysis was conducted using a Beckman Cytoflex S. In the competitive binding experiment, biotin-AAV2 or AAV843 particles at an equivalent epitope concentration of 200 nM (2 × 10^9^ vg/μL) were mixed with various concentrations of peptides (0–4,000 nM). After the mixture, incubation with 1 μL latex beads conjugated with mNAbs, IVIG or NAbs in 20 μL PBS at room temperature for 30 min, PE-Streptavidin (BD#349023) and incubated in the dark for an additional 30 min. Subsequently, 50 μL PBS was added, and analysis was conducted using a Beckman Cytoflex S. Percentage inhibition was calculated as the reduction in PE fluorescence intensity on antibody-conjugated beads in the presence of peptides relative to the no-peptide control, reflecting competition between peptides and biotinylated AAV particles for antibody binding.

#### Animal models

Female C57BL/6N mice (6–8 weeks, Beijing Hfkbio) were maintained under specific pathogen-free conditions.

##### AAV2 immunization model

Mice received i.v. injections of AAV2-CMV-GFP (5 × 10^7^ – 5 × 10^10^ vg), which is single-stranded (ss). Plasma was collected at day 14 for NAb titration and *in vitro* peptide inhibition assays.

##### Passive immunization model

C57BL/6N mice were injected i.v. with IVIG (Yeasen #36110ES80). After 24 h, plasma NAb levels were determined by standard neutralization assays.

##### Repeated-dosing gene therapy models

(1) Mice received ssAAV2-CMV-GFP (5 × 10^10^ vg) or IVIG (1 mg), followed 14 or 1 days later by peptide administration (i.v. or i.p.) and ssAAV2-CMV-Luc (1 × 10^11^ vg). Luc expression was measured at days 5 to day 25 post-injection using an IVIS system. (2) Plasma from four hemophilia B patients (26 weeks post BBM901 therapy) was used to purify IgG via Protein A/G columns. Each mouse received 1 mg of purified IgG (retro-orbital) followed by IdeS (Acro #IDS-S5143-2500U) after 1 h. After 12 h, AAV843 decoy peptides (10 μg) were injected (i.p.), and 4 h later, scAAV843-TTR-Gluc (5 × 10^11^ vg) was administered (i.v.). Gaussia Luc activity was measured 4 weeks later (Pierce #16160).

##### Peptide administration and pharmacodynamics

Mice received i.p. or i.v. injections of various peptide doses. Plasma was collected at 0, 0.5, 1, 4 and 6 h to evaluate NAb inhibition kinetics.

##### Safety assessment

Mice were injected (i.p.) with human-derived or viral-derived peptide mixtures, or OVA protein (InvivoGen #vac-pova). Plasma was collected at 24 h for cytokine analysis (BD #552364) and at day 7 for peptide- or OVA-specific antibody detection via direct ELISA.

### Statistical analysis

In addition to the statistical methods delineated in the AI algorithms, we employed the Student’s *t test* to compare independent single factors. Significant differences were indicated as ∗*p* < 0.05, ∗∗*p* < 0.01, and ∗∗∗*p* < 0.001. Each group comprised a minimum of four variables (biological replicates), and the mean and error values of each group were calculated, with each experiment conducted no less than three times.

## Data and code availability

The datasets and code of study are available at https://github.com/ZhxinMath/aav-nab-inhibitor-ai/tree/main. The version of the software in this study was illustrated at the beginning of the code. Additionally, the AI models and peptide analysis tools can be accessed and tested via the following website: http://www.siweiyu.top:7879/.

## Acknowledgments

This work was supported by grants from the Fundamental and Interdisciplinary Disciplines Breakthrough Plan of the 10.13039/501100002338Ministry of Education of China (JYB2025XDXM503 to Z.L.), 10.13039/501100010041Tianjin Municipal Science and Technology Commission grant (25ZXZSSS00510 to Z.L., 23JCZXJC00040 to X.P.), 10.13039/501100001809National Natural Science Foundation of China (82430010 to Z.L., 82470138 to X.F., and 82300159 to L.W.), CAMS Innovation Fund for Medical Sciences (CIFMS) (2024-I2M-ZH-016 to Z.L., 2025-I2M-XHZY-034 to Z.L.).

## Author contributions

X.P., B.X., D.Y., and R.F. performed experiments; X.P. and X.Z. built the AI models; T.B., Y.C., F.X., W.L., Y.C., R.Y., and X.L. collected the clinical samples; W.W., H.L., and Y.C. built the murine models; L.Z., X.P., and S.Y. designed the research and wrote the paper, all authors read and approved the final manuscript.

## Declaration of interests

The authors declare no competing interests.

## Supplemental information

Document S1. Figures S1–S11, Tables S2, and S3

Table S1. LoRA result on all AAV types

Document S2. Article plus supplemental information
