# Supplementary material for: Sequence-based artificial intelligence-guided inhibitory peptides to counteract AAV neutralizing antibodies in gene therapy
Source: Mol Ther Adv. 2026 Aug 6;34(4):201832. doi: 10.1016/j.omta.2026.201832 (PMC13572001; doi:10.1016/j.omta.2026.201832)
Supplement: Supplementary file 1 [file mmc1.pdf]

OMTA, Volume *34*

Supplemental information

Sequence-based artificial intelligence-guided inhibitory peptides to counteract AAV neutralizing antibodies in gene therapy

Bingqi Xu, Xin Zhang, Dandan Yu, Panjing Wang, Ying Chi, Wei Liu, Feng Xue, Lingling Chen, Huiyuan Li, Wentian Wang, Yunfei Chen, Xiaofan Liu, Rongfeng Fu, Renchi Yang, Wenwei Shao, Siwei Yu, Xiaolei Pei, and Lei Zhang

Supporting Information

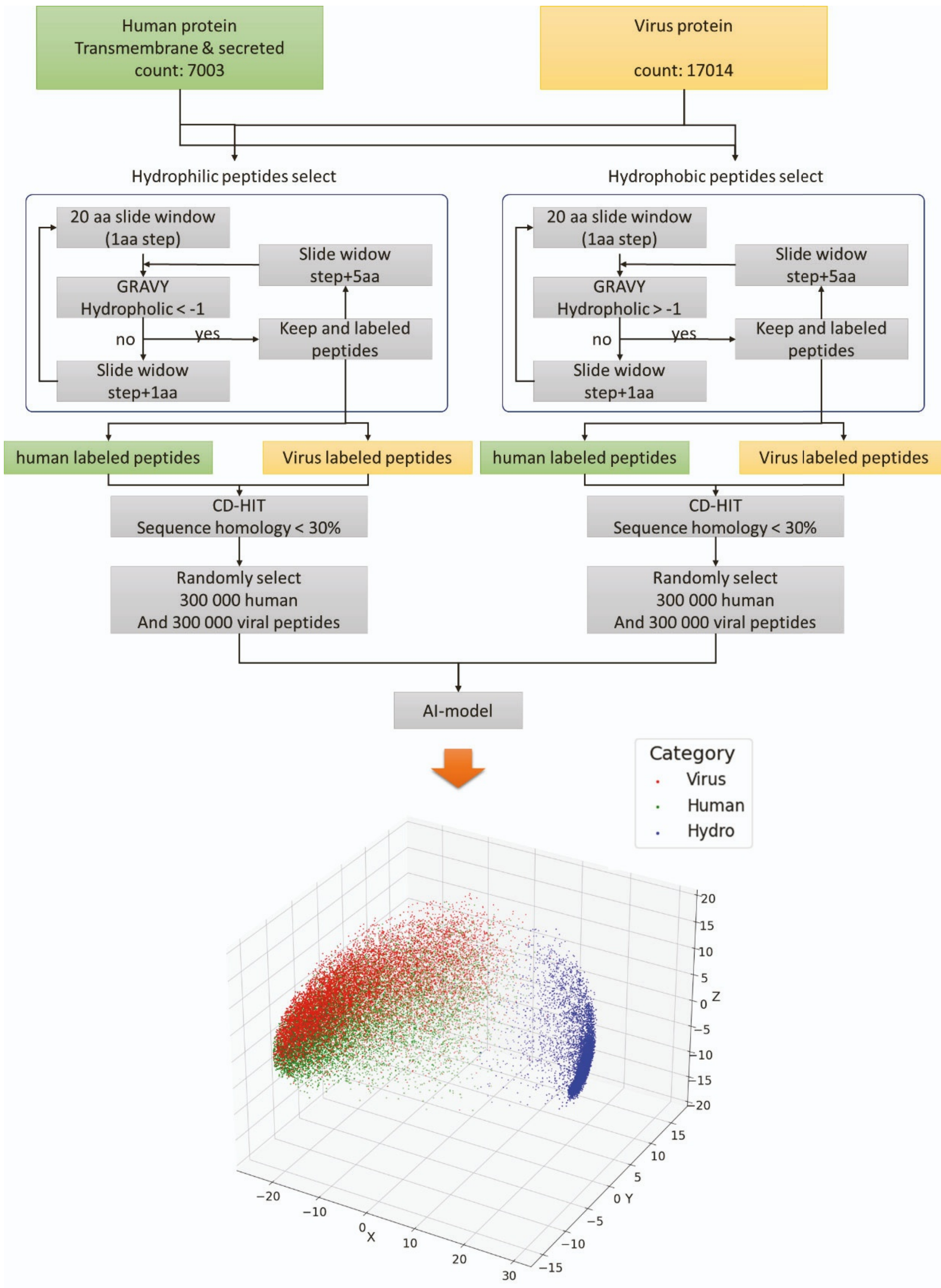

Figure S1. Enhanced model training strategy incorporating hydrophobic peptide classification. A third label, "hydrophobic peptide," was introduced into the training dataset

during the fine-tuning phase. The BERT model effectively distinguished among human-derived, virus-derived, and hydrophobic peptides, demonstrating improved classification capacity for diverse peptide types.

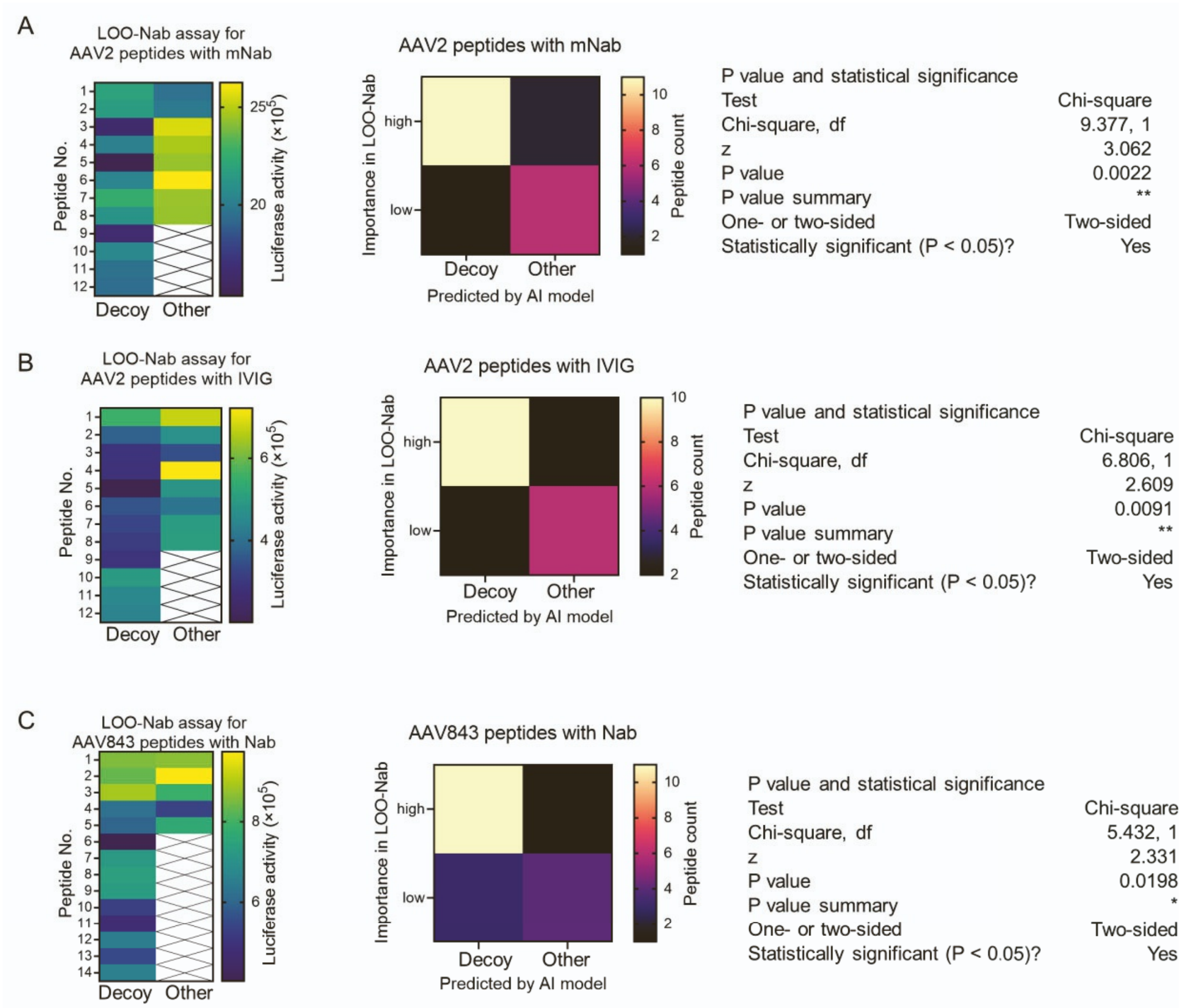

Figure S2. Evaluation of individual peptide contributions to NAbs inhibition using the Leave-One-Out (LOO) strategy. The inhibitory efficacy of peptide mixtures was assessed after sequential omission of individual peptides against: (**A**) mouse-derived NAbs induced by AAV2 immunization, (**B**) naturally occurring NAbs contained in IVIG, and (**C**) NAbs from hemophilia B patients following AAV843-based gene therapy. Peptides were categorized into effective and ineffective groups based on their impact on inhibition. A chi-square test was then performed to compare these classifications with predictions from the BERT model.

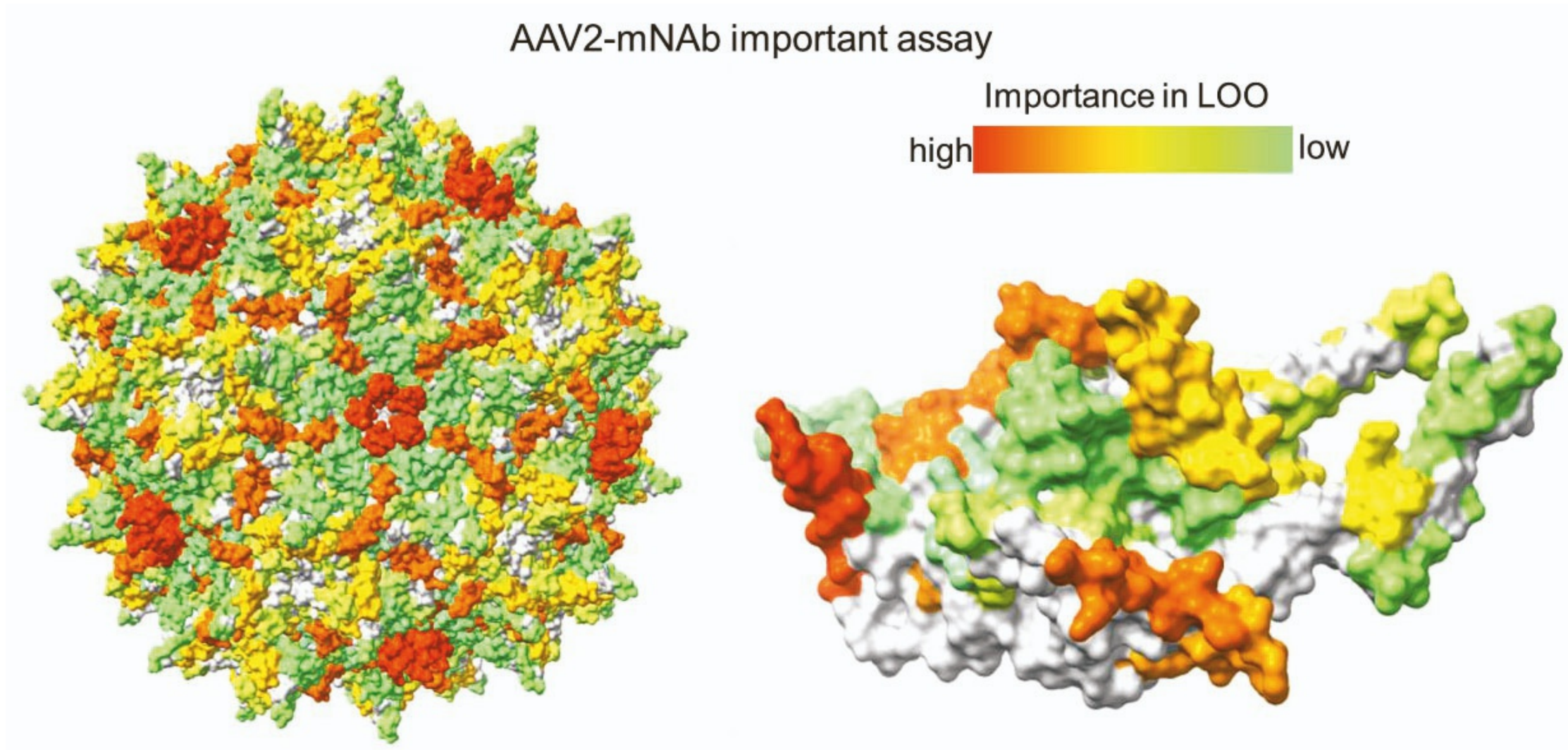

Figure S3. The spatial distribution and relative significance of decoy peptides on the three-dimensional AAV2 capsid structure (PDB: 6ih9), informed by Leave-One-Out neutralization assays. The maps peptides whose omission most significantly impairs neutralization by mouse-derived NAbs.

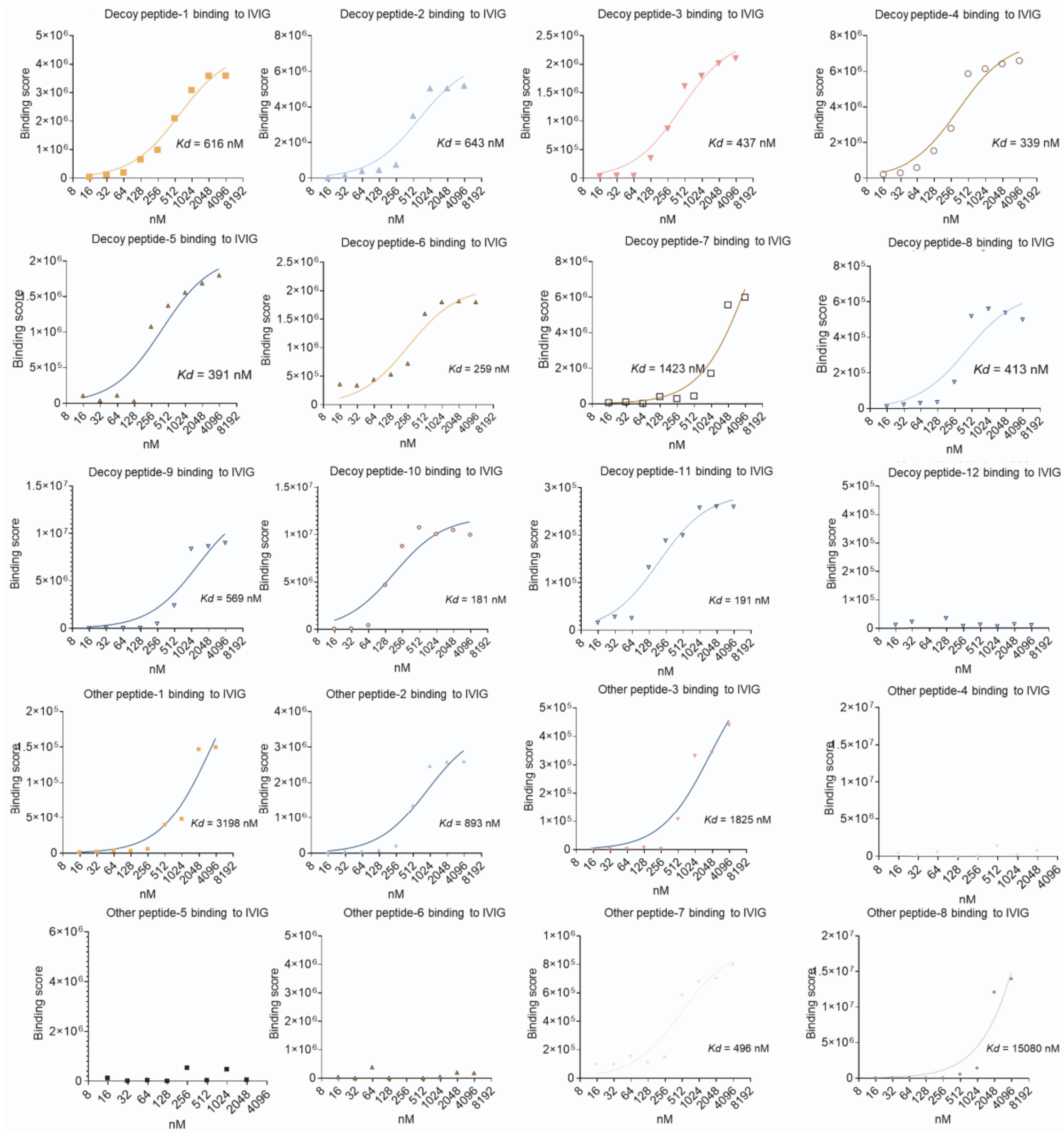

Figure S4. Saturation binding assay to assess the direct interaction between AAV2-derived decoy peptides and IVIG antibodies. Latex beads coated with IVIG were incubated with increasing concentrations of AAV2 decoy peptides. The binding affinity was evaluated by calculating the dissociation constant (*Kd*), reflecting the strength of peptide-antibody interactions.

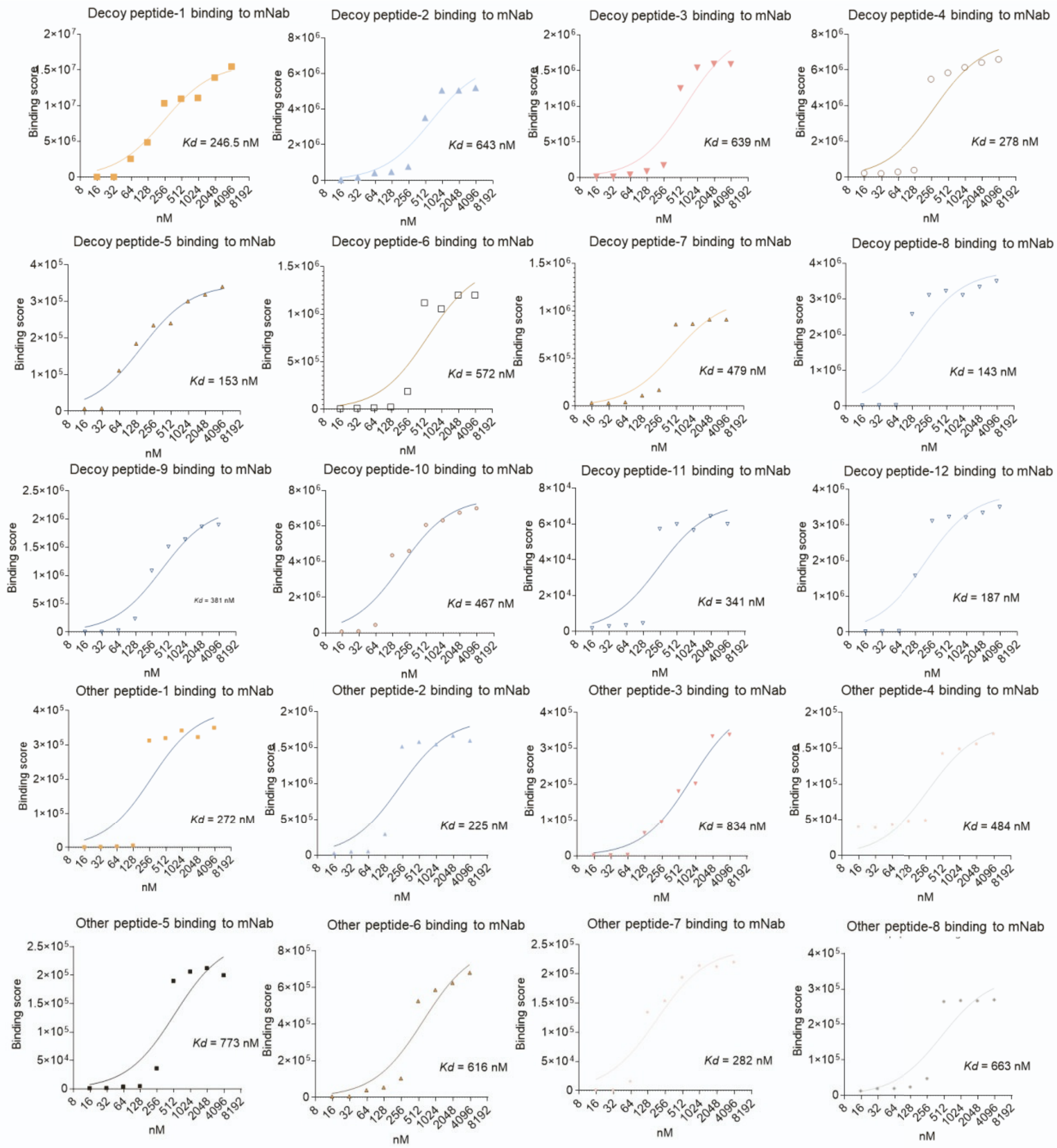

Figure S5. C57 mice were immunized via intravenous injection with AAV2-GFP to induce the production of anti-AAV2 neutralizing antibodies (mNAbs). One week post-immunization, venous blood plasma was collected, and immunoglobulins were purified using protein A/G affinity adsorption. The purified immunoglobulin (Ig) molecules containing mNAbs were conjugated to latex beads. Various doses of biotin-labeled AAV2 particles or peptides were then incubated with the mNAbs-conjugated latex beads. PE-streptavidin was used to detect the biotin signal. The fluorescence intensity of PE on the beads was measured to quantify the binding of peptides or AAV2 particles to mNAbs. A saturation curve was plotted, and the binding constant (*Kd*) was calculated.

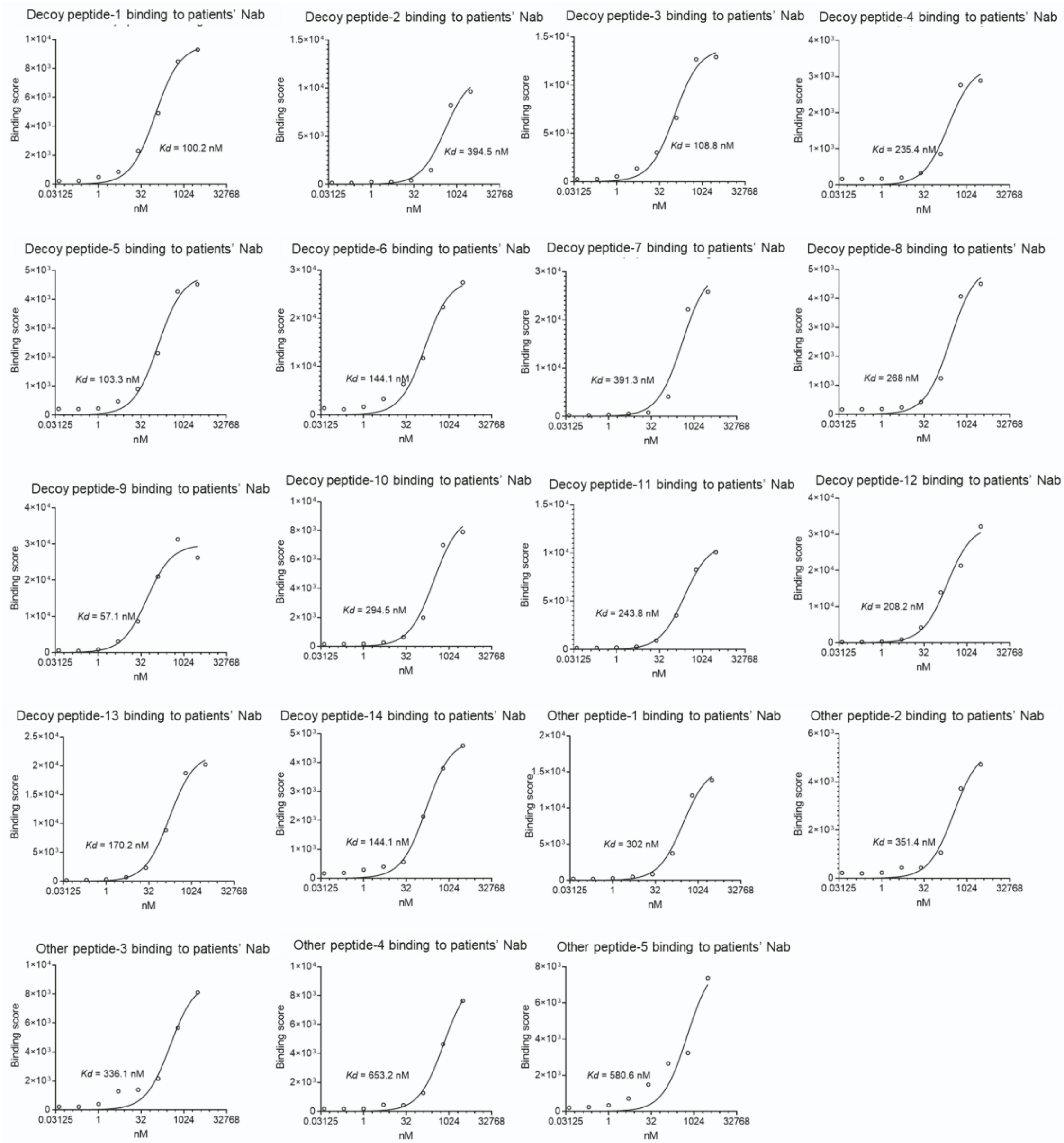

Figure S6. Saturation binding assays to evaluate the direct interaction between AAV843-derived decoy peptides and neutralizing antibodies (NAbs) from patient plasma. Latex beads coated with immunoglobulins from patients who received AAV843-based gene therapy were incubated with varying concentrations of decoy peptides. The binding affinity between the peptides and NAbs was assessed, and the dissociation constant (*Kd*) was calculated to quantify peptide-antibody interactions.

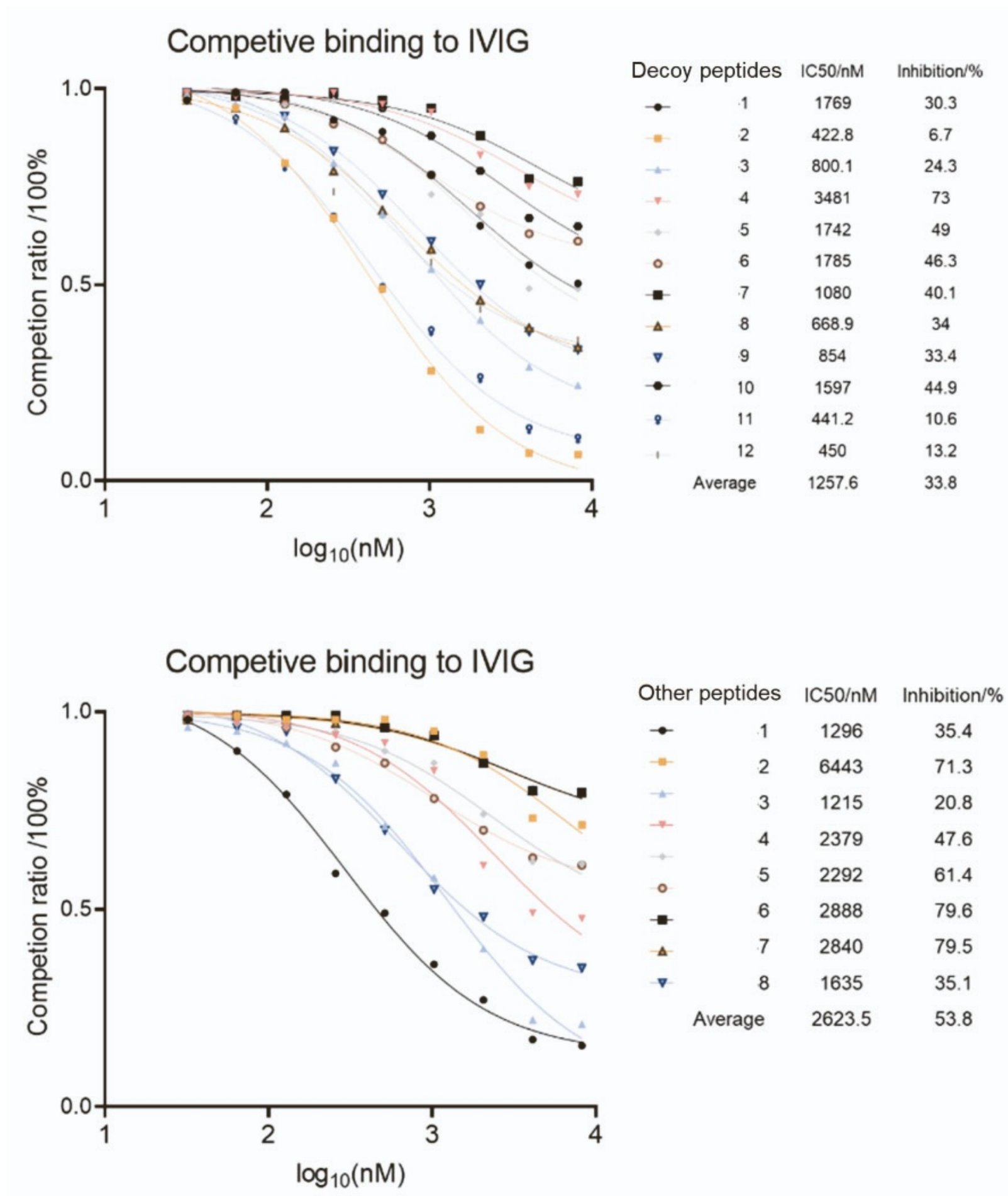

Figure S7. Biotin-labeled AAV2 particles, equating to a concentration of 200 nM, were combined with varied peptide concentrations (ranging from 0 to 8192 nM) and co-incubated with 1 μL of antibody-coated beads (IVIG). Post-PE-Streptavidin staining and flow cytometry, competitive binding curves and half-maximal inhibitory concentration (IC50) were determined.

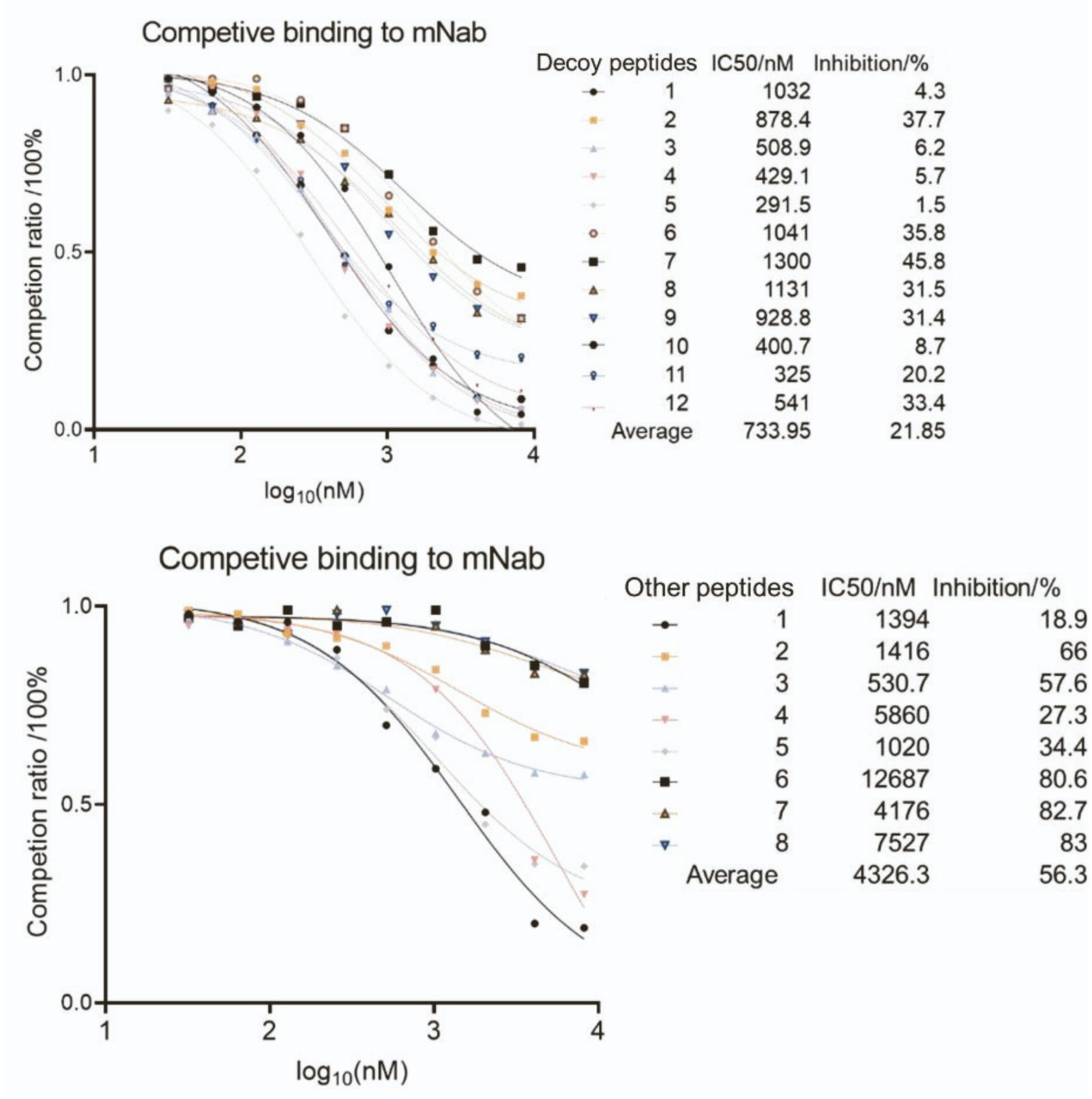

Figure S8. Biotin-labeled AAV2 particles, equating to a concentration of 200 nM, were combined with varied peptide concentrations (ranging from 0 to 8,192 nM) and co-incubated with 1 μL of mNAbs conjugated beads. Post-PE-Streptavidin staining and flow cytometry, competitive binding curves and half-maximal inhibitory concentration (IC50) were determined.

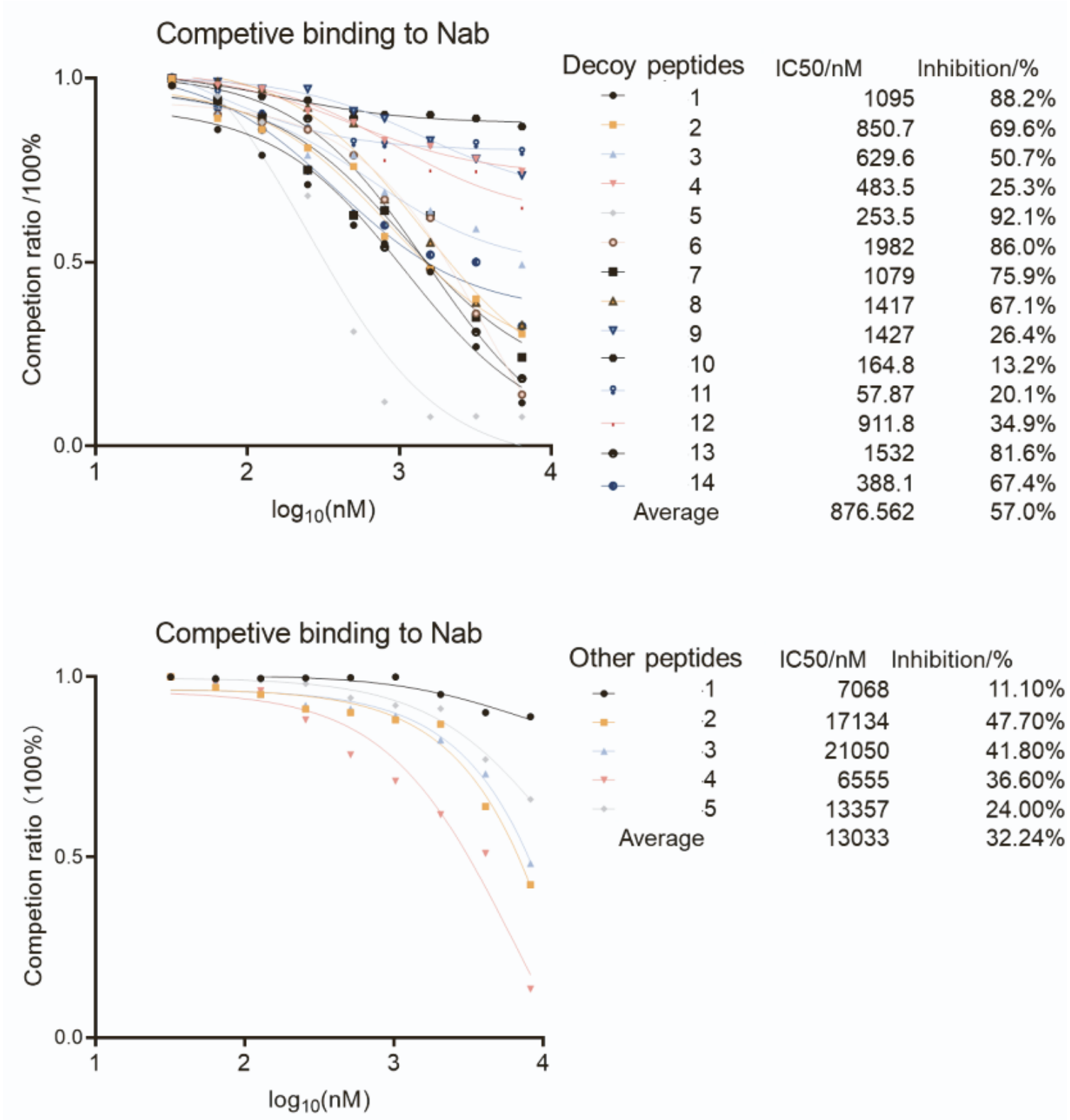

Figure S9. Biotin-labeled AAV843 particles, equating to a concentration of 200 nM, were combined with varied peptide concentrations (ranging from 0 to 8,192 nM) and co-incubated with 1 μL of beads-conjugated NAbs from the HB patients with gene therapy. Post-PE-Streptavidin staining and flow cytometry, competitive binding curves and half-maximal inhibitory concentration (IC50) were determined.

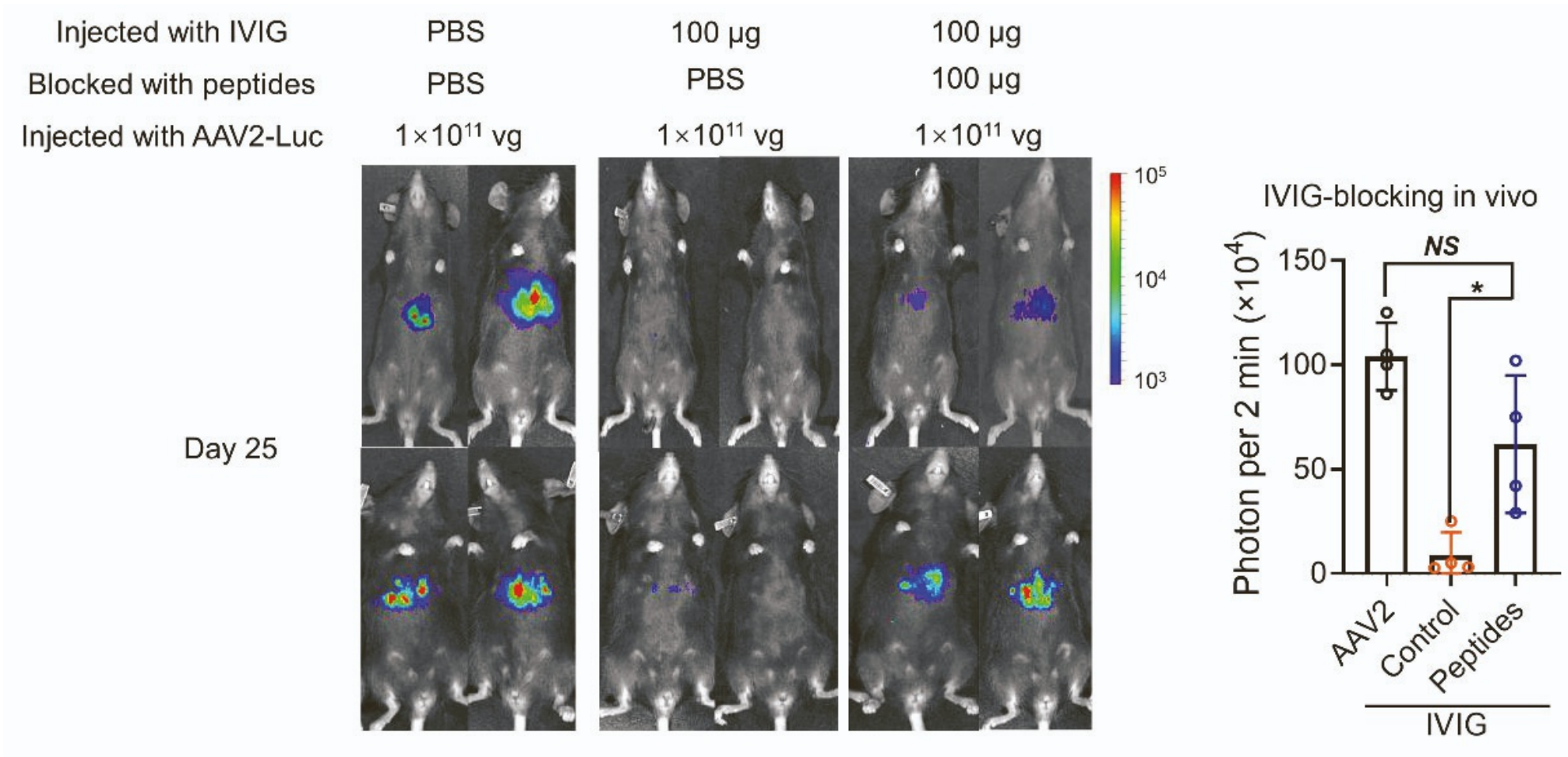

Figure S10. On the first day, IVIG (100 µg per mouse) or PBS was intravenously administered to C57 mice. On the secondary day, 100 µg of mixed peptides or solvent control was injected intraperitoneally. Four hours post-peptide injection, 1×10^{11} vg of AAV2-Luc was intravenously administered. On the twenty-fifty days post-secondary treatment, luciferase activity in mouse liver tissues was observed and quantified using IVIS imaging.

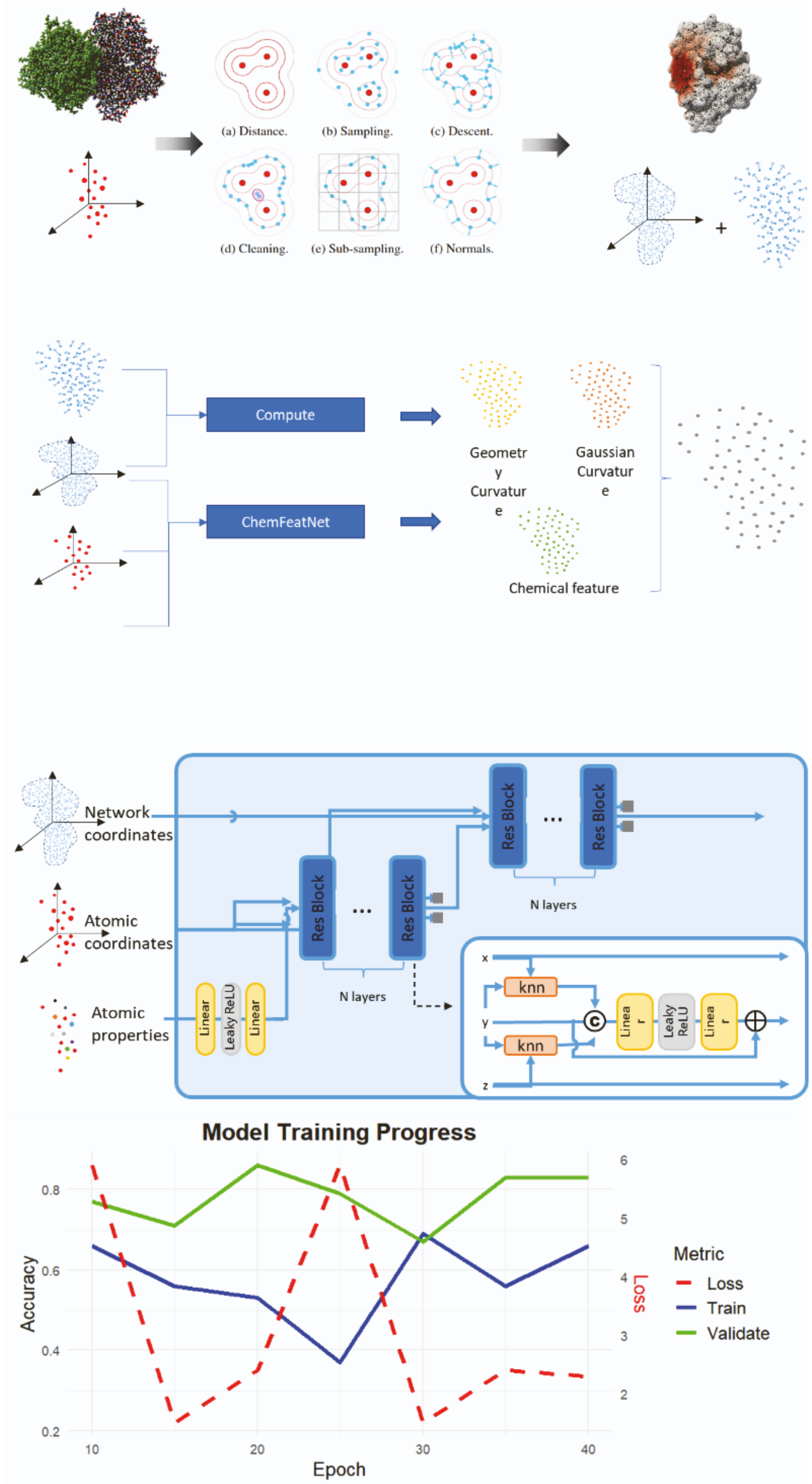

Figure S11. Using models, Masif the classification accuracy for identifying protein or protein surface-sourced patches from different species was found to be less than 0.8 in our study.

Table S1. LoRA result on all AAV types.

Table S2. AAV2 and AAV843 sourced peptides predicted by BERT model

Capsid	sequence	length	position_start	Prediction
AAV2	WALPTYNNHLYKQISSQSGA	20	247-	Other peptide-1
AAV2	QISSQSGASNDNHYFGYSTP	20	259-	Other peptide-2
AAV2	KEVTQNDGTTTIANNLTSTV	20	321-	Decoy peptide-1
AAV2	SAHQGCLPPFPADVFMVPQY	20	356-	Decoy peptide-2
AAV2	DVFMVPQYGYLTLNNGSQAV	20	368-	Decoy peptide-3
AAV2	PLIDQYLYYLSRTNTPSGTT	20	436-	Decoy peptide-4
AAV2	RTNTPSGTTTQSRLQFSQAG	20	447-	Decoy peptide-5
AAV2	QFSQAGASDIRDQSRNWLPG	20	461-	Other peptide-3
AAV2	PGPCYRQQRVSKTSADNNNS	20	479-	Other peptide-4
AAV2	SKTSADNNNSEYSWTGATKY	20	489-	Other peptide-5
AAV2	EYSWTGATKYHLNGRDSLVN	20	499-	Other peptide-6
AAV2	NPGPAMASHKDDEEKFFPQS	20	518-	Other peptide-7
AAV2	GKQGSEKTNVDIEKVMITDE	20	543-	Decoy peptide-6
AAV2	SEKTNVDIEKVMITDEEEIR	20	547-	Decoy peptide-7
AAV2	TEQYGSVSTNLQRGNRQAAT	20	573-	Decoy peptide-8
AAV2	STNLQRGNRQAATADVNTQG	20	580-	Decoy peptide-9
AAV2	VPANPSTTFSAAKFASFITQ	20	653-	Decoy peptide-10
AAV2	KRWNPEIQYTSNYNKSVNVD	20	693-	Other peptide-8
AAV2	TSNYNKSVNVDFTVDTNGVY	20	701-	Decoy peptide-11
AAV2	FTVDTNGVYSEPRPIGTRYL	20	712-	Decoy peptide-12
AAV843	WALPTYNNHLYKQISSASTG	20	248-	decoy-1
AAV843	YKQISSASTGASNDNHYFGY	20	258-	decoy-2
AAV843	NDNHYFGYSTPWGYFDFNRF	20	269-	decoy-3
AAV843	IQVKEVTTNDGVTTIANNLT	20	319-	decoy-4
AAV843	QYGYLTLNNGSQAVGRSSFY	20	376-	decoy-5
AAV843	YAHSQSLDRLMNPLIDQYLY	20	426-	decoy-6
AAV843	YLNRTQNQSGSAQNKDLLFS	20	446-	other-1
AAV843	RGSPAGMSVQPKNWLPGPCY	20	466-	decoy-7
AAV843	PGPCYRQQRVSKTKTDNNNS	20	481-	other-2
AAV843	KTDNNNSNFTWTGASKYNLN	20	494-	other-3
AAV843	ASKYNLNGRESIINPGTAMA	20	507-	other-4
AAV843	TAMASHKDDKDKFFPMSGVM	20	523-	decoy-8
AAV843	FGKESAGASNTALDNVMITD	20	544-	decoy-9
AAV843	NPVATERFGTVAVNLQSSST	20	571-	decoy-10
AAV843	VAVNLQSSSTDPATGDVHVM	20	581-	decoy-11
AAV843	PANPPAEFSATKFASFITQY	20	656-	decoy-12
AAV843	KRWNPEVQYTSNYARSANVD	20	694-	other-5
AAV843	YARSANVDFTVDNNGLYTEP	20	706-	decoy-13
AAV843	VDNNGLYTEPRPIGTRYLTR	20	716-	decoy-14

Table S3. AAV2 and AAV843 novel peptides designed by VAE

ID	seq	Length
AAV843_novel_011	MFMMPMHPPFHGHVHHVHVI	20
AAV843_novel_016	MVVVCFHPMVMPMHPGVHVI	20
AAV843_novel_613	MVEPFCVVHVVVWCVAIVVI	20
AAV843_novel_14	GKCAKHKNHHHCHHHHAKCG	20
AAV2_novel_413	IVYVIILMVIVQVVCVHVVI	20
AAV2_novel_711	MHHHHLHHFWFFHHVVVVIK	20
AAV2_novel_1015	MVVLVVGLLVVVVCVVEVEI	20
AAV2_novel_1216	IIIVCVQENNNEQQQQKVQI	20
AAV2_novel_1318	MFIVCQQQIQQFFWAIHQQI	20
AAV2_novel_1419	MVVVHVVHHHHVHHHVHHMI	20
